# Supplementary material for: How well do national and local policies in England relevant to maternal and child health meet the international standard for non-communicable disease prevention? A policy analysis
Source: BMJ Open. 2018 Nov 12;8(11):e022062. doi: 10.1136/bmjopen-2018-022062 (PMC6252630; doi:10.1136/bmjopen-2018-022062)
Supplement: Supplementary file 1 [file bmjopen-2018-022062supp001.docx]

Additional material

Appendix 1: The Policy Appraisal Framework used to analyse the policy documents identified

| **Priority Areas** | **Policy options** | **Detail** | **Search terms for data extraction** | **Inclusion and Exclusion criteria^a,b^** |
| --- | --- | --- | --- | --- |
| **Maternal health**  *Objective 3: Reducing modifiable risk factors*  *Overarching principle : life- course approach* | 1. Social media campaigns and/or dietary guidance | 39.8 | social media, nutrition, education, healthy eating, health promotion, social marketing, diet, food, guidelines, guidance, healthy lifestyle, mass media, campaigns, health promotion, eat well plate, 5 a day | **Inclusion:** Makes reference to dietary education and/or materials including dietary guidelines or intent to develop dietary guidelines. Includes material specifically for adolescent females (preconception health). Includes reference to external guidance linked in the policy document that meet the criteria, as long as the external guidance is accurately referenced in the correct place in the policy document.  **Exclusion**: Education initiative solely focusing on children’s diets, breastfeeding (except diets for breastfeeding women), alcohol consumption and smoking. |
|  | 1. Healthy food and health promotion in public institutions | 39.5, 39.9, 41.4 (iv) | public institute, standard, guideline, healthy environment, active transport, government agencies, food, nutrition, physical activity exercise, movement, school, hospital, meals | **Inclusion:** Reference to guidelines/health promotion of food, diet and physical activity health promoting environments within public institution. Includes reference to schools, hospitals and any government agencies.  **Exclusion:** Smoke-free, alcohol and breastfeeding environments. |
|  | 1. Economic tools to increase healthy and reduce less healthy food consumption | 39.6  30.2 | taxes, subsidies, sugar sweetened beverages, fiscal strategies, fruit and vegetable, finance, vouchers | **Inclusion:** Must make reference to intent to use fiscal measures to increase consumption of healthy foods and reducing consumption of less healthy foods. Includes pricing initiatives in schools, hospitals, leisure centres.  **Exclusion:** Physical activity or public transport subsidies. |
|  | 1. Nutrition labelling | 39.10 | food labelling, nutrition labelling, packaged food, front of pack, back of pack, traffic lights, health claims, nutrition information panel | **Inclusion:** Must make reference to nutrition information or improving health. Labelling on pre-packaged foods or nutrition facts. Includes nutrition prompts about foods provided by an establishment, e.g. catering in schools, hospitals, leisure centres. Nutrition labelling for infants and babies including baby formula is included.  **Exclusion:** Reference to food safety. |
|  | 1. Guidelines to reduce sodium, saturated fat, free sugar and increase fruit and vegetables for manufacturers to create healthy agriculture, retail and catering sectors | 39.3, 39.4, 39.7 | sodium, salt, trans (fat) saturated fat, (free) sugars, portion/serve size, fruit and vegetables, reformulation. Agriculture, farming, food retailers, caterers, subsidies, land availability, energy density | **Inclusion:** Product reformulation and the improvement of the nutritional quality of the food – specifically saturated/trans fat, sugars, salt, fruit and vegetables, energy density or portion sizes. Not required to directly refer to maternal health. Includes agricultural, manufacturing, retail and catering sectors. Includes reference to changing purchasing of a product with less salt, sodium, sugar, or fat of catering in schools, hospitals, leisure centres etc. |
|  | 1. Physical activity guidelines and/or public campaigns | 41.4 (iii), 41.4 (v), 41.5, 41.1 | mass media, physical activity, inactivity, sedentary, active transport, exercise, healthy lifestyle, mass media, social media, leaflet, booklets | **Inclusion:** Reference to guidelines/recommendations for physical activity, exercise and active transport or ways to reduce inactivity. Includes intent to develop physical activity guidelines. Includes material regarding adolescent females (preconception health).  **Exclusion:** Education initiatives solely focusing on children. |
|  | 1. Urban, transport and recreation planning to increase daily activity | 41.4 (i), 41.4 (iv) | urban planning, urban transport, recreation, infrastructure walking, cycling, safety, active transport, green space, natural environments, lockers, facilities, shower | **Inclusion:** Improving physical activity and active transport facilities including accessibility, availability, affordability and safety. Includes community playgrounds and outdoor spaces. Includes initiatives to encourage active transport leading to improved physical activity.  **Exclusion:** Initiatives to enhance social connectivity. |
| Urban Planning ^c^ | 1. Green Spaces and physical activity facilities | 41.4 (iv) | green Spaces, playgrounds, community gyms, physical activity, sports, parks | **Inclusion:** Increasing numbers or improving community green spaces and the availability of council-led physical activity facilities that enable people to be physically active. |
|  | 1. Active Transport | 41.4  41.4 (i) | traffic safety, walking cycling, footpath, pathway, active transport | **Inclusion:** Safety in terms of traffic lights and street lights and safety to enable active transport including footpaths and any sort of pathway and availability of community bikes. Explicit reference to changes at a local policy level that improve the opportunity for active transport, e.g. changes to infrastructure and physical environmental improvements. |
|  | 1. Licensing or Zoning of less healthy food outlets | 39.7  39.4 | zoning, takeaway outlets, diary, corner store | **Inclusion:** Licensing and zoning to regulate the density of less healthy food outlets. |
| **Infant health**  *Objective 3:*  *Reducing modifiable risk factors*  *Overarching principle : life- course approach* | 1. Breastfeeding guidelines/ initiatives | 39.1, 18.5, Annex 43(i) | breastfeed, nursing | **Inclusion:** Recommendations for breastfeeding and reference made to breastfeeding recommendations or action plans. Includes promotion of breastfeeding friendly environments and encouragement or promotion of breastfeeding. Includes cross-reference to breastfeeding guidelines and links to reputable sources with explicit mention of the criteria.  **Exclusion:** Food and dietary guidelines for breastfeeding women. |
|  | 1. Complementary feeding guidelines/ initiatives | 39.1 | complementary feeding, introducing solids, weaning | **Inclusion:** Reference to recommendations/ guidelines for complementary feeding and/or introducing solids, including infant and toddlers dietary guidelines. |
|  | 1. Guidelines/ restrictions for the marketing of food and beverages to children | 33, 38, 39.2, 43.1 | marketing, advertising, promotions, sponsorship | **Inclusion:** Details/restrictions of food marketing or advertising to children or reference made to the need for food marketing/advertising guidelines. Includes television, online, sponsorship of sports, school activities and other recreation activities. Also includes marketing not necessarily targeted at children but is intended for them, e.g. baby formula marketing. |
|  | 1. Physical activity opportunities in education institutions | 41.4 (ii) | physical education, active movement, walking , cycling, physical activity, exercise, active transport, preschools, day care, Kindergartens, child care, outdoor play | **Inclusion:** Reference made to guidelines/ recommendations to physical activity options for education institutions. Includes requirements for making changes to environment to encourage physical activity. Includes initiatives external to school grounds but school-driven such as ‘walking school bus’. Needs to refer to education institutions including pre-schools through to universities.  **Exclusion:** Initiatives solely focusing on staff of these institutions. |
| **Strengthening health systems**  *Objective 4*  *Overarching principle: Empowerment of people and communities* | 1. Improving the knowledge, skills and motivation of the workforce (maternal and infant health professionals) through training in NCD and obesity prevention, specifically regarding behaviour change, diet and physical activity | 48.4 (i, ii),  48.3(vii  30.8 | skills, development, education, workforce develop, knowledge, training, behaviours skills | **Inclusion:** Regards training of health professionals inclusive of maternal and child health professionals to increase knowledge and skills in behaviour change, diet and/or physical activity requirements, inclusive of mothers (preconception, pregnancy and breastfeeding) and infants. Is about NCD prevention.  **Exclusion:** Does not include smoking or alcohol consumption. |
|  | 1. Maternal and Infant health professionals empower people to manage their own condition and support family-centred/self-care, particularly among high-risk populations. | 48.3 (vi),,  49.4 (iii) | empower, Patient/family/ community centre, self-care, cultural competent, cultural awareness, minority groups, low-income groups | **Inclusion:** Reference to training of health professionals inclusive of maternal and child health professionals in skills that ensure the level of care is patient/family centred and/or empowering or a particular consideration for high risk populations. Not just about training but also competencies and training to adequate levels to provide empowerment. Includes empowerment of people who help target group, e.g. carers, parents.  **Exclusion:** General person-centred approach statements. |
| **Evidenced based strategies**  *Overarching principle* | 1. Adoption of evidence based practice | 18.7  32.1  39.8  41.5 | best practice, evidence, strategies, evidence informed/based | **Inclusion:** Must refer to the use of evidence-base strategies (latest scientific evidence and/or best practice) to inform the development and implementation of policies/programmes /interventions relevant to diet and physical activity. |
|  | 1. Strengthen evidence base for effective dietary and physical activity interventions through research and evaluation | 53.5  41.6 | research, evaluation, scientific, knowledge base, best practice | **Inclusion:** Refers to research and evaluations of interventions/programmes to improve diet and/or physical activity. Includes the documents referring to the need for review or development guidance. |
| **Multisectoral action**  *Objective 2*  *Overarching principle* | 1. Multisectoral development of the policy | 18.4  27  29  30.5  30.6 | *searched the policy authors and the introduction of the policy* | **Inclusion:** Reference to the process of policy development. Must refer to more than one government department/government funded institution in policy development. |
|  | 1. Multistakeholder collaboration and cooperation | 21.3  39.3  41.3 | collaboration, co-operation, engagement, stakeholder, community, local government | **Inclusion:** Development of collaborations /engagements with stakeholders for interventions/programme/policies. Includes stakeholders from multiple sectors, such as NGOs, industry, civil society groups, professional bodies, different disciplines, etc.  **Exclusion:** Not required to be directly related to diet/food and physical activity behaviour change. Documents cannot simply include multistakeholder action as a throwaway comment but must name examples. |
| **Governance and accountability**  *Objective 2*  *Overarching Principle: Management of real, perceived or potential conflicts of interest* | 1. Management or acknowledgement of conflicts of interest | 38  18.9 | conflicts of interest, conflicting industries/ bodies, funding sources, guidelines | **Inclusion:** Reference to the explicit awareness of managing conflicts of interest when developing diet, physical activity or food/physical activity environment initiatives.  **Exclusion:** Initiatives related to smoking and alcohol consumption. |
|  | 1. Set targets/ establish accountability frameworks for NCDs, diet or physical activity behaviours | 29  30.7  Pg. 5. Voluntary Global Targets | target, framework, accountability, plan, KPI's, indicators, outcomes, outputs, goal, objective, vision | **Inclusion:** Must refer to a framework or targets set by WHO or national/local governments that relate to NCDs, obesity, diet and physical activity. Includes intention to develop a framework or targets.  **Exclusion:** Accountability frameworks for policies that do not explicitly relate to NCD prevention i.e. professional code of conduct accountability frameworks. |
|  | 1. Implement participatory, community based approaches | 30.10  48.1 (ii) | participation, community, empowerment, involvement, capacity building, consultation. | **Inclusion:** Reference to community members/ representative groups involvement in the development/ implementation of interventions/programmes and policy development. Must recognise that the community have a key role to play, not just about guidelines but how the local community are involved. |

^a^ It is acceptable for links to external documents to be included as a positive result for the different policy options. However the external documents must be linked to in the body of the text of the policy document being appraised and must the body of text must explicitly mention the policy option area being explored.

^b^ Focusses on the physical components of health related to physical activity and diet, therefore doesn’t include mental health in this instance.

^c^ This section only applies to the Local policies from the case study.

^d^ The colouring corresponds to the ecological model of health levels: light grey is individual behaviour change, grey is supportive social and physical environments in institutions, and dark grey is macro environmental policy.
