# Supplementary material for: How well do national and local policies in England relevant to maternal and child health meet the international standard for non-communicable disease prevention? A policy analysis
Source: BMJ Open. 2018 Nov 12;8(11):e022062. doi: 10.1136/bmjopen-2018-022062 (PMC6252630; doi:10.1136/bmjopen-2018-022062)
Supplement: Supplementary file 2 [file bmjopen-2018-022062supp002.pdf]

Additional material

Appendix 2: List of all policy documents appraised arranged by policy type

**Policy Document Title**	**Date Published**	**Author**
Acts and codes		
The Processed Cereal–Based Foods and Baby Foods for Infants and Young Children (England) Regulations	December 2003	Department of Health
The Childcare Act 2006	July 2006	Ministry of Justice
The Infant Formula and Follow-on Formula (England) Regulations	December 2007	Department of Health
The UK Code of Non-Broadcast Advertising, Sales Promotion and Direct Marketing	August 2010	Advertising Standards Authority<br>Ofcom
The UK Code of Broadcast Advertising (BCAP code)	August 2010	Advertising Standards Authority<br>Ofcom
Health and Social Care Act 2012	June 2012	Department of Health
Children and Families Act 2014	March 2014	Department for Education<br>Ministry of Justice<br>Department for Business, Innovation & Skills[a]<br>Department for Work and Pensions<br>Department of Health
The Food Information Regulations 2014	July 2014	Department for Environment, Food and Rural Affairs
White papers		
Healthy Weight Healthy Lives	January 2008	Department of Health<br>Department for Children, Schools and Families[b]
Healthy Lives, Brighter Futures: the strategy for children and young people's health	February 2009	Department of Health<br>Department for Children, Schools and Families[b]
Healthy Lives Healthy People: our strategy for public health in England	November 2010	Department of Health
Health Visitor Implementation Plan 2011-2015: a call to action	February 2011	Department of Health
Supporting Families in the Foundation Years	August 2011	Department for Education<br>Department of Health
Joint Strategic Needs Assessment and Joint Health and Wellbeing Strategies Explained	December 2011	Department of Health
National Planning Policy Framework	March 2012	Department for Communities and Local Government

An Update in the Government's Approach to Tackling Obesity	July 2012	National Audit Office
Improving Children And Young People's Health Outcomes: a system wide response	February 2013	Department of Health
Giving all Children a Healthy Start in life	March 2013	Department for Education<br>Department of Health
Developing a Specification for Lifestyle Weight Management Services	March 2013	Department of Health
National Health Visitor Plan: progress to date and implementation 2013 onwards	June 2013	Department of Health<br>NHS England<br>Public Health England<br>NHS Health Education England
Briefing on the Government's Ambition for Cycling	August 2013	Department for Transport
Moving More, Living More: Olympic and Paralympic Games legacy	February 2014	Cabinet Office<br>Department of Health<br>Department for Digital, Culture, Media and Sport,<br>Department for Education<br>Mayor of London
Maternity and Early Years: making an good start to family life	March 2015	Department of Health
The Governments Responsibility Deal	March 2015	Department of Health
Statutory Guidance on Joint Strategic Needs Assessments and Joint Health Wellbeing Strategies.	March 2015	Department of Health
A Plan for Public Procurement: food & catering - balanced scorecard for public food procurement	July 2015	Department for Environment, Food and Rural Affairs
Five Year Forward View	October 2015	NHS England
Public Health Outcomes Framework, 2013-2016 (1A and 1B)	November 2015	Department of Health
Childhood Obesity: a plan for action	August 2016	Department of Health
Soft Drinks Industry Levy	December 2016	HM Revenue and Customs<br>HM Treasury
Evidence-based guidance documents		
Postnatal Care up to 8 Weeks After Birth	July 2006	NICE
Obesity Prevention	December 2006	NICE
Food Standards Agency Nutrient and Food Based Guidelines for UK Institutions	October 2007	Food Standards Agency

Behaviour Change: general approaches	October 2007	NICE
Antenatal Care for Uncomplicated Pregnancies	March 2008	NICE
Everybody Active, Every Day: an evidence-based approach to physical activity	October 2014	Public Health England
The Link Between Pupil Health and Wellbeing and Attainment: a briefing for head teachers, governors and staff in education settings	November 2014	Public Health England
Behaviour Change: individual approaches	January 2015	NICE
Physical Activity: for NHS staff, patients, and carers.	March 2015	NICE
Weight Management Before, During and After Pregnancy	July 2015	NICE
Postnatal Care	July 2015	NICE
Maternal and Child Nutrition	July 2015	NICE
A Quick Guide to the Government's Healthy Eating Recommendations	August 2015	Public Health England
Obesity in Children and Young People: prevention and lifestyle weight management programmes	August 2015	NICE
Multiple Pregnancy: twin and triplet pregnancies	September 2015	NICE
Sugar Reduction: the evidence for action	October 2015	Public Health England
From Evidence into Action: opportunities to protect and improve the nation's health	October 2015	Public Health England
Obesity and the Environment: regulating the growth of fast food outlets.	November 2015	Public Health England<br>Local Government Association
Nutrition Support in Adults	November 2015	NICE
The Influence of Maternal, Foetal and Child Nutrition on the Development of Chronic Disease in Later Life	November 2015	Scientific Advisory Committee on Nutrition
Obesity in Adults: pre obesity in adults: prevention and lifestyle weight management lifestyle weight management programmes	January 2016	NICE
Building the Foundations: tackling obesity through planning and development	February 2016	Local Government Association<br>Town and Country Planning Association<br>Public Health England

Healthier Food Procurement	August 2016	Local Government Association
Antenatal Care	September 2016	NICE
Childhood Obesity: brave and bold action	November 2016	House of Commons Health Committee
Guide to Creating a Front of Pack (FoP) Nutrition Label for Pre-packed Products Sold Through Retail Outlets	November 2016	Department of Health<br>Food Standards Agency<br>Welsh Government<br>Northern Ireland Government<br>Food Standards Scotland<br>British Retail Consortium
Healthy Weight, Healthy Futures: local government action to tackle childhood obesity	February 2017	Local Government Association
Local Policies		
Local Transport Plan 3: strategy and implementation plan for Southampton.	March 2011	Southampton City Council
Supporting Carers in Southampton: a joint strategy for improvement 2011-2015	December 2012	Southampton City Council<br>Southampton City Clinical Commissioning Group
Southampton's Joint Health and Wellbeing Strategy: gaining healthier lives in a healthier city	January 2013	Southampton City Council<br>Southampton City Clinical Commissioning Group
Southampton Local Plan: compliance with national planning policy framework	June 2013	Southampton City Council
Southampton City Strategy 2014-2025	July 2014	Southampton City Council
Southampton Safe City Strategy 2014-17	July 2014	Safe City Partnership (Hampshire Constabulary, Southampton City Council and other representatives)
Our Vision for Future Southampton	June 2015	Southampton City Council
Health and Wellbeing Strategy 2017-2025	March 2017	Southampton City Council<br>Southampton City Clinical Commissioning Group
Cycling Southampton: a cycling strategy for our city 2017-2027	June 2017	Southampton City Council
Children and Young People's Healthy Weight Plan	June 2017	Southampton City Council

[a] superseded by Department for Business, Energy and Industrial Strategy, [b] superseded by Department of Education
