# Supplementary material for: How well do national and local policies in England relevant to maternal and child health meet the international standard for non-communicable disease prevention? A policy analysis
Source: BMJ Open. 2018 Nov 12;8(11):e022062. doi: 10.1136/bmjopen-2018-022062 (PMC6252630; doi:10.1136/bmjopen-2018-022062)
Supplement: Author's manuscript [file bmjopen-2018-022062.draft_revisions.pdf]

BMJ Open is committed to open peer review. As part of this commitment we make the peer review history of every article we publish publicly available.

When an article is published we post the peer reviewers' comments and the authors' responses online. We also post the versions of the paper that were used during peer review. These are the versions that the peer review comments apply to.

The versions of the paper that follow are the versions that were submitted during the peer review process. They are not the versions of record or the final published versions. They should not be cited or distributed as the published version of this manuscript.

BMJ Open is an open access journal and the full, final, typeset and author-corrected version of record of the manuscript is available on our site with no access controls, subscription charges or pay-per-view fees (http://bmjopen.bmj.com).

If you have any questions on BMJ Open's open peer review process please email info.bmjopen@bmj.com

BMJ Open

How well do national and local policies in England relevant to maternal and child health meet the international standard for non-communicable disease prevention? A policy analysis

Journal:	*BMJ Open*
Manuscript ID	bmjopen-2018-022062
Article Type:	Research
Date Submitted by the Author:	30-Jan-2018
Complete List of Authors:	Penn-Newman, Daniel; University of Southampton, MRC Lifecourse Epidemiology Unit<br>Shaw, Sarah; University of Southampton, MRC Lifecourse Epidemiology Unit<br>Congalton, Donna; University of Auckland, Liggins Institute<br>Strommer, Sofia; University of Southampton, MRC Lifecourse Epidemiology Unit<br>Rose, Taylor; University of Southampton, MRC Lifecourse Epidemiology Unit<br>Lawrence, Wendy; University of Southampton, MRC Lifecourse Epidemiology Unit<br>Chase, Debbie; Southampton City Council, Public Health<br>Cooper, Cyrus; University of Southampton, MRC Lifecourse Epidemiology Unit; NIHR Southampton Biomedical Research Centre, University Hospital Southampton NHS Foundation Trust<br>Barker, Mary; University of Southampton, MRC Lifecourse Epidemiology Unit; NIHR Southampton Biomedical Research Centre, University Hospital Southampton NHS Foundation Trust<br>Baird, Janis; University of Southampton, MRC Lifecourse Epidemiology Unit<br>Inskip, Hazel; University of Southampton, MRC Lifecourse Epidemiology Unit; NIHR Southampton Biomedical Research Centre, University Hospital Southampton NHS Foundation Trust<br>Vogel, C; University of Southampton, MRC Lifecourse Epidemiology Unit
Keywords:	Health policy < HEALTH SERVICES ADMINISTRATION & MANAGEMENT, maternal health, child health, Nutrition < TROPICAL MEDICINE, physical activity, PUBLIC HEALTH

SCHOLARONE™
Manuscripts

How well do national and local policies in England relevant to maternal and child health meet the international standard for non-communicable disease prevention? A policy analysis

Daniel Penn-Newman[1], Sarah Shaw[1], Donna Congalton [2], Sofia Strommer [1], Taylor Rose[1], Wendy Lawrence[1,3], Debbie Chase[4], Cyrus Cooper[1,3], Mary Barker[1,3], Janis Baird[1], Hazel Inskip[1,3], Christina Vogel[1]

[1] Medical Research Council Lifecourse Epidemiology Unit, University of Southampton, Southampton General Hospital, Tremona Road, Southampton, SO16 6YD, UK

[2] Liggins Institute, University of Auckland, Level 2, Building 505, 85 Park Rd, Grafton, Auckland 1142, NZ

[3] NIHR Southampton Biomedical Research Centre, University Hospital Southampton NHS Foundation Trust and University of Southampton University Hospital Southampton NHS Foundation Trust and University of Southampton, Southampton SO16 6YD, UK

[4] Public Health, Southampton City Council, Civic Centre, Southampton SO14 7LY, UK

Correspondence to: Christina Vogel, cv@mrc.soton.ac.uk, Tel: 023 8076 4042, University of Southampton, Southampton General Hospital, Tremona Road, Southampton SO16 6YD, UK

Word count: abstract: 211, main text: 4893

For peer review only

Abstract

Objective: To quantify the extent to which national policies for England, and local policies for Southampton City, that are relevant to maternal and child health meet the international standards for nutrition and physical activity initiatives that are set out in the World Health Organisation Global Action Plan for the Prevention and Control of NCDs (WHO Action Plan).

Design: The policy appraisal process involved three steps: i) identifying policy documents relevant to maternal and infant health from , ii) developing a policy appraisal framework for the WHO Action Plan, and iii) analysing the policies using the framework.

Setting: England and Southampton City

Participants: 57 national and 10 local policies

Results: Evidence-based practice was the most frequently implemented policy option (79% national, 80% local). The least frequently implemented policy options among the national policies were complementary feeding (9%), managing conflicts of interest (12%) and workforce empowerment skills (14%). Physical activity related policy options were better implemented than dietary options among the local policies. Macro-environmental policy options received a lower priority than organisational or individual options among national policies but higher priority among local policies.

Conclusions: Further action is needed to manage conflicts of interest and implement policy options that promote a system-wide approach to challenging NCDs caused by poor diet and physical inactivity.

Key words: policy, maternal health, child health, nutrition, physical activity, public health

For peer review only

Strengths and limitations of this study

- This is the first polity review to focus on maternal, infant and child health for non-communicable disease prevention and considers government activity at both national and local levels
- The assessment method reflects adherence to the World Health Organisation international best practice and enable easy identification of areas for improvement
- Use of a second assessor and verification by policy makers provided quality assurance
- Policies from only one local authority were assessed as a case study
- The use of a dichotomous measure does not provide comprehensive details about the scale of policy implementation

peer review only

1. Introduction

Noncommunicable diseases (NCDs) including cardiovascular disease and diabetes are the primary cause of death globally.[1] In the UK, they account for 89% of all adult deaths.[2] Exposure to risk factors such as obesity, physical inactivity and poor diet begins early, before conception and in utero.[3] Accordingly, optimising maternal, infant and child health is a top priority on the international health agenda. There is thus a need to align the NCD prevention agenda with the high level directives encouraging member states to enact nutrition initiatives targeting preconception, pregnant and breastfeeding mothers, and the first 1000 days.[4-6]

National and local governments' policies play a key role in leading action on NCD prevention. National governments are best placed to monitor population behaviours and health indicators, and have a responsibility to enact policies that will optimise the health of their citizens.[7] Local governments have the ability to work with the community, respond to local needs and create local settings that support healthier lifestyle behaviours, particularly in the UK where public health responsibilities were conferred on local authorities in 2012.[8]

For peer review only - http://bmjopen.bmj.com/site/about/guidelines.xhtml

National government policies cover a variety of document types and can be categorised as: i) acts and codes - legally binding approved by the House of Commons and Lords that are implemented by an appropriate government agency [9], ii) white papers – an outline of government's strategic direction or priorities for action on a particular subject [10], and iii) evidence-based guidelines – implicit policies including summaries of evidence into advice on best-practice standards or recommended course of action, such as guidance by the National Institute for Health and Care Excellence (NICE).[11] The content of local and national policies may vary considerably or may be closely aligned, depending on whether there is strong national leadership on an issue or high heterogeneity in local authorities' structures and priorities.[12] Similarly, different types of national policies may vary in scope or represent comprehensive, complementary action depending on a government's political ideology, time in office and power across the parliamentary houses, amongst other factors.[13]

To strengthen state members' efforts to address the growing global burden of NCDs, the 66th World Health Assembly endorsed the World Health Organisation (WHO) Global Action Plan for the Prevention and Control of NCDs (Action Plan) and NCD Global Monitoring Framework in 2013.[14] The Action Plan recognises the primary role of governments in responding to the growing challenge of NCDs, highlights the links between poor-diet, physical inactivity and NCDs, and states the importance of enhancing maternal and child health. The Action Plan set out a range of policy options that governments at all levels can implement and an international standard against which government action can be assessed. The policy options work across the ecological model of health [15] by targeting: i) individual-level behaviour change, ii) organisational-level determinants including social and physical settings, and iii) macro-environmental-level determinants including fiscal policy and

For peer review only - http://bmjopen.bmj.com/site/about/guidelines.xhtml

infrastructure. Multi-component policy action that works across these levels has shown to be more effective at improving lifestyle behaviours than action at a single level,[16] though it is recognised that macro-environmental policy action is politically challenging.[7]

The current NCD Global Monitoring Framework measures progress in reducing prevalence rates of health outcomes or risk factors but does not comprehensively assess progress on policy options to improve both dietary and physical activity behaviours.[17] Other policy appraisal frameworks focus on national government progress for a single behaviour and have not considered policy options specific to maternal, infant and child health.[13, 18] The 2014 report of the Chief Medical Officer highlighted that half the population of women aged 25-34 years in England are overweight or obese and noted the need to improve dietary and physical activity behaviours among women of reproductive age.[19] An understanding of how national and local government policies are currently supporting women and children to adopt healthy lifestyles is needed to identify gaps for further government leadership. Therefore, this study aimed to quantify the extent to which English national and local government policies that are relevant to maternal and child health meet the international standard set out in the WHO Action Plan. The specific aims of this study were to i) identify national government policies, and local government policies using Southampton City as a case study, ii) analyse the existence of policy in implementation against the Action Plan policy options, and iii) assess variations implementation of the policy options by policy type (local/national and acts/white papers/evidence-based guidance), and the three levels of the ecological model of health.

3. Methodology

For peer review only - http://bmjopen.bmj.com/site/about/guidelines.xhtml

The policy appraisal process involved three main actions: i) identify national policies for England and local policies for Southampton City relevant to maternal and child health or dietary and physical activity behaviours, ii) develop a policy appraisal framework based on the WHO Action Plan policy options, and iii) analyse the identified policies using the appraisal framework. Ethical approval was not required for this secondary analysis of publicly available information.

3.1 Identification of National and Local Policy Documents

The policies were identified in December 2015 and an updated search was conducted in June 2017. Government websites were searched for acts and codes, briefing notes, white papers and evidence-based guidelines. A range of websites were searched including the Department of Health, Department for Education, Department for Children Schools and Families, and the Department for Communities and Local Government. Additionally, executive agency websites, including the Food Standards Agency, Public Health England, NICE and the Local Government Association, were searched because these agencies can influence policy development. Policies focusing on disease treatment or specific nutrients, or those detailed in policies published at a later date were excluded. The compilation of the identified national policies was then sent to a programme manager at Public Health England for verification and identification of missing policies. Documents published during the UK Coalition and Conservative Governments, from May 2010 to June 2017, were the focus of this review. Key policies prior to this date were included if they: i) informed current policies, or ii) had not been superseded.

Southampton City was used as a case study for the local policy analysis. Southampton is a large city on the south coast, more deprived than other cities in the affluent South East of England. Southampton is ranked the 67th most deprived of the 326 Local Authorities in

For peer review only

England,[20] and in 2014/15, 47% of women in Southampton were classified as overweight or obese at their maternity booking appointment.[21] To aid methodological rigor and completeness, the identification of local Southampton policies was supported and verified by a public health consultant (DChase) at the Southampton City Council. Policies were extracted from the Southampton City Council website or provided by City Council staff.

3.2 Development of a Policy Appraisal Framework

Three researchers (CV, DCongalton, DPN) independently identified components of the WHO Action Plan relevant to maternal and child health, and dietary or physical activity behaviours that could be categorised into priority areas for the appraisal framework. Each researcher then highlighted policy options from the WHO Action Plan that were relevant for maternal (preconception, pregnancy, lactation and motherhood) and/or infant and child health. The policy options were specific initiatives that could be identified in policies. All policy options were independent from each other and corresponded with a single priority area. Supplementary material Appendix 1 presents the final appraisal framework agreed upon by the three researchers. Discrepancies were resolved by returning to the WHO Action Plan to ensure consistency and accuracy.

A total of six priority areas were established for the appraisal framework. These included i) maternal health, ii) infant/child health, iii) strengthening health systems, iv) evidence-based strategies, v) multisectoral action, and vi) governance and accountability. Each priority area had between two and seven accompanying policy options. A total of 20 policy options were included in the national policy appraisal framework. The same 20 were included in the local policy appraisal framework plus three additional urban planning policy options to encompass local government jurisdiction over planning. To ensure consistency in identifying the

For peer review only

presence or absence of a policy option within a policy key search terms and inclusion and exclusion criteria for each policy option were created (supplementary material Appendix 1).

The policy options were categorised into three levels in line with the ecological model of health: individual, organisational and environmental (supplementary material Appendix 1). Seven policy options could not be categorised according to the three levels of the ecological model because they were not action-orientated but represented good public health processes. These process-orientated policy options are necessary for governments to produce high-quality, intensive policies to address NCDs, for example adopting an evidenced-based approach, managing conflicts of interests and establishing accountability frameworks.

3.3 Policy Appraisal Process

One researcher (DPN) assessed all identified policies against the appraisal framework. Policies were reviewed individually and search terms were used to pinpoint relevant sections that were comprehensively assessed to determine whether or not the policy option was present. The inclusion and exclusion criteria for each policy option were used to guide this procedure. Random selections, comprising 44% of the national policies and 50% of the local policies, were double coded by a second researcher (SShaw). Inconsistencies in coding were found in 4% of the double coded policies, indicating over 95% level of agreement. Discrepancies were discussed with a third researcher (CV) and agreement was reached after returning to the policy and appraisal framework.

3.4 Statistical analysis

Descriptive analyses were used to describe the policies by type, author, and date of publication. The frequency for each of the policy options was calculated to ascertain their representation across the national and the local policies. Frequencies of policy options were

For peer review only

also calculated according to the three national policy types: acts and codes, white papers and evidence-based guidelines. Cochran's Q test was used to test for differences in policy option frequency across the three ecological levels (individual, organisational, and environmental) to identify whether there was difference in policy option implementation according to level of action. All statistical analyses were conducted using Stata statistical software package version 13.[22]

4. Results

4.1 Characteristics of Identified Policies

A total of 57 national policies relevant to maternal and child health were identified; 47 were published between May 2010 and June 2017, and 10 prior to 2010. Figure 1 provides a schematic representation of the national policy identification process.

Evidence-based guidance documents (n=27, 47%) and white papers (n=22, 39%) represented the vast majority of identified policies, with acts and codes representing the remainder (n=8, 14%). Supplementary material Appendix 2 details the title, publication date and authors of each identified policy according to the three policy types. Of the 28 organisations that authored the national policies, the Department of Health was most prolific, authoring more than one third of all policies (n=21, 37%; Table 1), including half of the acts (n=4, 50%) and almost three quarters of the white papers (n=16, 73%). NICE was the second most common author (n=14, 25%), publishing the majority of the evidence-based guidance documents (n=14, 52%). Most policies were authored by a single organisation (n=46, 81%). However, 14 organisations were only co-authors. The Department of Health was the most common co-author (8 of the 11 co-authored policies).

For peer review only

Table 1: List of authors for identified national policies

Author	Number of single authored documents	Number of co-authored documents	Total[a] n (%)
Department of Health	13	8	21 (37)
National Institute for Health Care Excellence	14	0	14 (25)
Public Health England	5	3	8 (14)
Department of Education[b]	0	6	6 (11)
Local Government Association	2	2	4 (7)
Advertising Standards Authority/ Ofcom	2	0	2 (4)
Department for Communities and Local Government	1	1	2 (4)
Department for the Environment, Food and Rural Affairs	2	0	2 (4)
Food Standards Agency	1	1	2 (4)
Ministry of Justice	1	1	2 (4)
National Health Service (NHS) England	1	1	2 (4)
British Retail Consortium	0	1	1 (2)
Cabinet Office	0	1	1 (2)
Department for Business, Innovation and Skills[c]	0	1	1 (2)
Department for Digital, Culture, Media and Sport	0	1	1 (2)
Department for Work and Pensions	0	1	1 (2)
Department of Transport	1	0	1 (2)
Food Standards Scotland	0	1	1 (2)
HM Revenue and Customs	0	1	1 (2)
HM Treasury	0	1	1 (2)
House of Commons Health Committee	1	0	1 (2)
Mayor of London	0	1	1 (2)
National Audit Office	1	0	1 (2)
NHS Health Education England	0	1	1 (2)
Northern Ireland Government	0	1	1 (2)
Scientific Advisory Committee on Nutrition	1	0	1 (2)
Town and Country Planning Association	0	1	1 (2)
Welsh Government	0	1	1 (2)

[a] column total reflects the total number of national policy documents published by each author and rounded percentage of all national policies (n=57) [b] Department for Children, Schools and Families superseded by Department of Education, [c] Department for Business, Innovation and Skills superseded by Department for Business, Energy and Industrial Strategy

For peer review only

Ten local policies were identified for appraisal, published between March 2011 and June 2017. The policies were authored by three different organisations (supplementary material Appendix 2). The Southampton City Council led or was involved in the development of all local policies. Southampton City Clinical Commissioning Group co-authored three policies (30%) and Southampton Safe City Partnership authored one (10%).

4.2 Policy appraisal findings - national

Figure 2 shows the frequency of the priority areas and policy options across the 57 national policies. The priority areas that support public health processes were frequently implemented. The evidence-based practice and strengthening the evidence base through research and evaluation policy options featured in 79% and 61% of the national policies respectively. Community participation and accountability frameworks both featured in 56% of the national policies. However, managing conflicts of interest was the second least frequent policy option (12%). Multi-stakeholder collaboration was mentioned in more than half (58%) of the national policies but multisectoral policy development was less common (19%).

The action-orientated priority areas were less frequently implemented than those supporting good public health processes, with child health having the lowest prevalence (Figure 2). Fewer than half (47%) of the policies included one of the four infants/children policy options. Complementary feeding initiatives was the least common policy option, appearing in only 9% of national policies, while children's food marketing restrictions featured in 16%. More than three quarters (81%) of the policies contained one of the seven maternal policy options. Maternal nutrition related policy options (19-46%) were better represented than those relating to maternal physical activity (26-28%). One third (35%) of policies contained strategies to strengthen health systems. The need for workforce development in behaviour change

For peer review only - http://bmjopen.bmj.com/site/about/guidelines.xhtml

strategies was cited in 32% of policies but workforce development in empowerment approaches was rarely mentioned (14%).

4.2.1 Policy content according to policy type

The frequencies of each policy option for the three national policy types are presented in Figure 3. Among the eight **acts and codes**, nutrition labelling was the most common policy option appearing in 50%. Restrictions on marketing foods and beverages to children and evidence-based practice featured in 38%, while product reformulation, managing conflicts of interest and multisectoral collaboration were mentioned in 25%. Six policy options did not feature in any of the acts or codes. The vast majority of the 22 **white papers** included evidence-based practice and community participation (both 82%) and 73% featured accountability frameworks and multisectoral collaboration. While nutrition in public institutions was the most common action-orientated policy option (50%), developing workforce empowerment skills was not cited in any white papers and complementary feeding guidance was mentioned in only one (5%). Most of the 27 **evidence-based guidance** documents included evidence-based practices (89%) and strengthening the evidence base through research (74%). Nutrition guidelines featured in almost two thirds (63%) but managing conflicts of interest was mentioned in only one (4%).

4.2.2 Policy content according to ecological model of health

Policy options to improve the macro environment, including economic tools and food marketing restrictions, were featured in half of the national policies (58%). Policy options to create supportive social and physical environments in organisations featured more frequently (65%), while those targeting individual level behaviour change were most common (72%). Cochran's Q test revealed that there were no statistically significant differences (p=0.1) in representation of the ecological levels across the national.

For peer review only

4.3 Policy appraisal findings - local

Figure 4 shows the frequency of each policy option (including the three additional urban planning policy options) across the 10 local policies. Most of the local policies cited evidence-based practice (80%), multisectoral collaboration (70%) and multisectoral policy development (50%). Maternal physical activity related policy options had a strong representation with physical activity guidance, urban planning, improving green spaces and physical activity facilities, and improving active transport infrastructure all appearing in 50% of the local policies. Nutrition-focused policy options featured less frequently with five maternal nutrition options featuring in 10% or less of the local policies. The two workforce development policy options also featured in only 10% of local policies. Analysis against the ecological model of health revealed that macro environmental policy options featured in 90% of the local policies, those targeting individual level behaviour change appeared in 50%, and those supporting healthy organisational settings in 30%. Cochran Q test revealed that there was a statistically significant difference in representation of these ecological levels across the local policies (p=0.02).

For peer review only

5. Discussion

5.1 Summary of Results

Among the 57 national and 10 local policies identified in this study, policy options supporting public health processes were implemented more frequently than the action-orientated options targeting maternal and child dietary and physical activity behaviours. This finding was consistent across the three types of national policies and the local policies, although some differences were apparent. Multisectoral policy development was more

common among the local than the national policies, while using targets to monitor policy implementation was more frequent in the national policies. Managing conflicts of interest was rarely considered in the white papers or evidence-based guidelines but was addressed more often in the acts and codes, and local policies.

For the action-orientated policy options, those with a nutrition focus were more frequent than the physical activity ones among the national policies. Acts and codes in particular did not contain any physical activity policy options. The opposite was observed for local policies where there was a heavy focus on urban planning strategies to promote physical activity. Complementary feeding guidance was virtually missing from all types of national policies, while nutrition labelling did not appear in any local policies. The need for workforce development in behaviour change skills was recognised across the white papers, evidence-based guideline and local policies, but workforce training in empowerment or self-care approaches was only cited in evidence-based documents and one local policy.

5.2 Comparison with Literature

This policy review is unique, compared with previous assessments of government action on obesity and NCDs in its focus on policy options specific to maternal, infant and child health. Existing policy frameworks consistent with the WHO Action Plan consider the entire population and measure progress for a specific behaviour or health outcomes. For example, the World Cancer Research Fund International NOURISHING framework and the International Network for Food and Obesity Research, Monitoring and Action Support (INFORMAS) Healthy Food Environment Policy Index (Food-Epi) considered policy activities for healthy eating and food environments,[23] while the WHO Global Monitoring Framework focuses on the prevalence of NCD risk factors.[17] Implementation of policy options that support the adoption of healthy nutrition and physical activity behaviours prior to

For peer review only

conception (including adolescence) and during pregnancy, infancy and childhood have been recognised by the UK Chief Medical Officer as critical to prevent NCDs in future generations and help curb healthcare costs.[19]

The results of this study identified that nationally, policy options related to maternal health were more frequently implemented than those related to child and infant health. General nutrition guidance and nutrition labelling were among the most frequent action-oriented policy options across the three types of national policies. Research from Thailand and England that used the Food-Epi framework similarly found good implementation of dietary guidelines and nutrition labelling when compared with international examples of good practice.[24, 25] However, our study additionally assessed the implementation of complementary feeding guidelines which we found to be the least well implemented policy option. Complementary feeding is important for child development, growth and health, and this period of life presents a window of opportunity to prevent malnutrition in all forms.[26] Over the past few decades progress on the implementation of policies to promote breastfeeding has been made but this has not been matched by efforts to support complementary feeding, as shown by our study's results. Other countries have also shown limited success in meeting WHO complementary feeding recommendations,[27, 28] indicating that government action on this issue is needed.

A systematic review of the determinants of poor nutrition in children aged below 3 years identified that effective intervention strategies include improving maternal self-efficacy, being of sufficient intensity and reaching mothers during pregnancy.[29] Building the capacity of health professionals through workforce development is thus essential to engagement and supporting behaviour change.[30] A review of policy options to address obesity in Spain identified workforce development in obesity prevention as one of the most popular approaches for government to adopt.[31] The results of our policy review showed

For peer review only - http://bmjopen.bmj.com/site/about/guidelines.xhtml

that workforce development in behaviour change skills was being implemented in England, but recognition of the need to up-skill healthcare professionals in empowerment approaches that improve self-efficacy was not included in any white papers, acts or codes. There is growing evidence that information is not enough to prompt behaviour change, particularly among disadvantaged groups, and that media campaigns increase inequalities in health behaviours.[32-34] While a number of the evidence-based documents reviewed in this study did include workforce empowerment approaches, the lack of coverage in white papers indicates that evidence is not adequate for policy option adoption.

Recent reviews of obesity-related policy analyses identified an overwhelming focus on national or state/provincial governments, and a lack of consideration for how policy option implementation varies between national and local governments.[13, 18] While limited to only one local authority, our study identified a clear difference in the focus of national and local policies. National documents contained a greater number of nutrition-related policy options, while local documents covered more policy options for physical activity, particularly macro-environmental options. The transition of public health into local authorities in the UK has provided increased opportunities for health considerations in planning and environment policies.[35] The dominance of physical activity over dietary policy options in Southampton mirrors attitudes across other local councils in England. A consultation with staff from 14 councils identified low awareness of policy options for creating healthy food environments, but good knowledge of planning activities to improve cycling and walking, and access to open spaces.[36] These findings suggest that the conditions for policy action, including public and political value, resources and operational capabilities.[37] are each being met for physical activity strategies but not yet for population level nutrition strategies.

There is a call for governments to enact regulation to create healthy food and physical activity environments as these policy options are consistently effective at improving lifestyle

For peer review only

choices and do not widen inequalities in the way that information strategies can.[16] Our study showed that policy options to improve the macro environment were less well implemented by the English government than those targeting organisational settings or individual level behaviour change, although this difference was not statistically significant. Reviews that assessed international actions to address obesity across low, middle and high income countries similarly identified limited appetite from national governments for regulatory action and a preference for educational strategies.[38, 39] The introduction of a UK sugar-sweetened beverage levy in 2018 indicates a growing global trend, led by action from governments in Mexico, France and Denmark, for stronger government actions to address obesity. However, concerns about adequate enforcement and the effectiveness of self-regulatory and voluntary codes, such as those used to govern restrictions for online advertising of unhealthy food to children or the reformulation of sugar and salt in foods, indicate that tougher regulatory action in these areas is still needed.[38]

Similar to reviews of the implementation of national policies related to food environments in New Zealand, Thailand and England,[24, 25, 40] we found that policy options supporting sound public health processes were implemented well across policy types. However, this study additionally identified that only a small proportion of English policies contained details about managing conflicts of interest. This issue is important for governments to consider during policy development and implementation because strategies to improve maternal and child dietary and physical activity behaviours could impact upon a number of profit-driven commercial industries. In particular, commercial interests from infant formula manufacturing companies directly compete with breastfeeding.[41] Strategies to manage conflicts of interest were not included in a number of policies where potential conflicts are likely including The Processed Cereal–based Foods and Baby Foods for Infants and Young Children (England)

For peer review only - http://bmjopen.bmj.com/site/about/guidelines.xhtml

Regulations. Limiting commercial influence and developing clear processes for accountability of both the public and the private sector are necessary.[23]

5.3 Implications for policy and research

The UK Government's current childhood obesity strategy outlined few measures for early childhood despite the WHO's Commission on Ending Childhood Obesity recommending member states develop clear complementary feeding guidance, including portion sizes and recommendations to avoid sugar sweetened beverages and energy-dense nutrient-poor foods. The much-awaited review by the Scientific Advisory Committee on Nutrition on complementary feeding will create an opportunity for the publication of clear guidelines in national white papers and their implementation by local and national agencies.

Health and social care services that support behaviour change by empowering individuals to generate their own solutions have shown beneficial effects on the self-efficacy of women from disadvantaged populations.[42] Practitioners from a range of backgrounds can be successfully trained in empowerment skills and these offer great potential to be used in routine health and social care to support behaviour change among harder to reach women and families.[43] The NHS 'making every contact count' movement recognises these opportunities for contact but further leadership is required from national government to include this policy option in white papers and systematically build workforce capacity in empowerment skills across England.

Leadership and funding from state and national governments has been shown to affect policy priorities at the local level. Local governments in the State of Victoria in Australia have higher implementation of policy initiatives to support physical activity and healthy eating than other states. This increased activity has been directly attributed to the mandatory requirement under the Victorian Health Act 1958 and the Planning and Environment Act

For peer review only

1987 for local governments to prepare Municipal Public Health Plans, in addition to the offer of financial incentives.[12, 44] In the UK, The National Planning Policy Framework requires planning departments to promote healthy communities, taking into account and supporting local Joint Health and Wellbeing Strategies. This national leadership has shown to be beneficial for physical activity related policy options but local public health teams now need to work strategically with planning and environment colleagues to support more healthy eating opportunities including zoning restrictions of takeaways or licensing requirements for the sale of healthy foods.[36] Collaborations between local universities and public health teams could provide necessary evidence for local government action to support development of healthy food environments, as local evidence provides impetus for local government action [12, 45]. Similarly, supranational unions, such as the European Union (EU), have provided leadership or hindrance for member states directing initiatives related to the analysis conducted in this study. While it was beyond the scope of this study to assess EU governance over English policies, Brexit provides opportunities for brave policy change that are supportive of healthy dietary and physical activity behaviours.

Recent evidence from a leaked email shows that Coca Cola placed indirect pressure on governments through an industry-funded scientific institute to influence policy to benefit commercial companies rather than the public.[46] Public confidence can be damaged if government's management of conflicts of interest is not sufficiently documented.[47] By using the international best practice standards for managing conflicts of interest, governments in England could appropriately manage this issue going forward.[48]

5.4 Strengths and Limitations

This study is one of few policy reviews to focus on maternal, infant and child health for NCD prevention and to consider government activity at both the national and local levels. The

For peer review only

study involved a comprehensive desktop search for three types of policies that was aided by senior health officials in local and national government to enhance completeness of the search strategy. Strengths of the appraisal framework include that it mirrored current international best practice standards, was developed and tested by three researchers independently, and included strict inclusion and exclusion criteria to guide data extraction. For quality assurance, one author completed the data extraction for all identified policies and half were also coded by a second researcher.

This study also had a number of limitations. Some policies may have been missed because they were not available electronically or were not found, and the local policy results for Southampton may not be representative of action in other local authorities. In addition, the appraisal framework and data extraction results were not validated through consultation with policy makers and other expert stakeholders due to resource limitations. Furthermore, the use of a dichotomous measure does not provide comprehensive details about the scale of policy option implementation and not all policy options would be expected to appear in every policy. However, this policy assessment method reflects overall adherence to the WHO international best practice, allows easy identification of areas for improvement and may be a useful for national governments in England and other countries to monitor progress on NCD prevention targeting women and children.

6. Conclusion

This study assessed policies relevant to improving maternal and child dietary and physical activity behaviours against the WHO international standard to understand the national and local progress in England of implementing policy options to prevent NCDs. Analyses of 57 national policies highlighted greater implementation of policy options related to maternal

For peer review only

than child health (particularly complementary feeding initiatives) and individually focused policy options (such as nutrition labelling) than environmental strategies. Action at the local level showed a stronger focus on policy options for physical activity than those for healthy eating, and for child health than for maternal health. Policy options that underpin good public health processes such as evidence-based strategies and multisectoral collaboration were widely adopted by both national and local governments, but further action is needed by policy makers to manage conflicts of interest and curb industry lobbying during policy development and implementation. Policy decision-making results from a combination of factors including the framing of an issue, political ideology, evidence of effectiveness and cost-effectiveness, timing and 'groundswell' or societal attitudes.[12, 13] Opportunities exist for the research community and health professionals to influence policy in a meaningful way by forming coalitions to mobilise societal support and framing issues as solutions in a way that is palatable to policy makers. The timing of external events can positively influence policy decision making. The 2012 Olympics provided impetus for policy action supportive of physical activity.[49] Challenging as the management of Brexit is, it provides a unique opportunity for policy change that holds great potential to reform food and transport systems to be supportive of healthy dietary and physical activity behaviours.

For peer review only

Acknowledgements

We are grateful to Patsy Coakley for computing support. We thank our funders for their support and would like to acknowledge that the views expressed in this publication are those of the authors and not necessarily those of the funding organisations.

Source of funding

This work was supported by funding from:

- NIHR Southampton Biomedical Research Centre, University of Southampton University Hospital Southampton NHS Foundation Trust
- European Union's Seventh Framework Programme (FP7/2007-2013), project EarlyNutrition (289346)
- CV was supported by a fellowship from the University of Southampton (PCTA36/2015)

Conflicts of interest statement

DPN, SShaw, DCongalton, DChase, HMI and CV have no conflicts of interest to declare. SStrommer, TR, WL, MB and JB have received grant research support from Danone Nutricia Early Life Nutrition. CC has has received consultancy, lecture fees and honoraria from AMGEN, GKS, Alliance for Better Bone Health, MSD, Eli Lilly, Pfizer, Novartis, Servier, Medtronic and Roche. The study described in this manuscript is not related to these conflicted relationships.

Contributor statement

DPN and CV conceived the study and wrote the first draft of the manuscript. HMI, JB, MB, DChase, CC and WTL contributed to the study design and helped draft the manuscript. DPN, TR, SStrommer and DChase identified the relevant policies. DPN, DCongalton and CV designed the policy audit tool. DPN, SShaw, DCongalton and CV conducted the policy audit. All authors read and approved of the manuscript.

Data sharing statement

The data are available upon request to the corresponding author.

For peer review only

References

1. Abajobir AA, Abate KH, Abbafati C, Abbas KM, Abd-Allah F, Abdulle AM, Abera SF, Aboyans V, Abu-Raddad LJ, Abu-Rmeileh NME *et al*: **Global, regional, and national comparative risk assessment of 84 behavioural, environmental and occupational, and metabolic risks or clusters of risks, 1990-2016: a systematic analysis for the Global Burden of Disease Study 2016**. *The Lancet*, **390**(10100):1345-1422.
2. **Noncommunicable Diseases (NCD) Country Profiles - United Kingdom** [http://www.who.int/nmh/countries/gbr_en.pdf]
3. Barker DJ, Thornburg KL: **Placental programming of chronic diseases, cancer and lifespan: a review**. *Placenta* 2013, **34**(10):841-845.
4. United Nations Secretary-General: **Global Strategy for Women's, Children's and Adolescent's Health (2016-2030)**. In. New York; 2015.
5. World Health Organisation: **Comprehensive Implementation Plan on Maternal, Infant and Young Child Nutrition**. In. Geneva, Switzerland; 2014.
6. World Health Organisation: **Report of the Commission on Ending Childhood Obesity**. In. Geneva, Swtizerland; 2016.
7. Swinburn BA, Sacks G, Hall KD, McPherson K, Finegood DT, Moodie ML, Gortmaker SL: **The global obesity pandemic: shaped by global drivers and local environments**. *Lancet* 2011, **378**(9793):804-814.
8. Department of Health UK: **Healthy Lives, Healthy People: Our strategy for public health in England**. In. London; 2010.
9. **Acts** [http://www.parliament.uk/about/how/laws/acts/]
10. **White Papers** [http://www.parliament.uk/site-information/glossary/white-paper/]
11. **What we do** [https://www.nice.org.uk/about/what-we-do]
12. Allender S, Gleeson E, Crammond B, Sacks G, Lawrence M, Peeters A, Loff B, Swinburn B: **Moving beyond 'rates, roads and rubbish': How do local governments make choices about healthy public policy to prevent obesity?** *Australia and New Zealand Health Policy* 2009, **6**:20.
13. Clarke B, Swinburn B, Sacks G: **The application of theories of the policy process to obesity prevention: a systematic review and meta-synthesis**. *BMC Public Health* 2016, **16**(1):1084.
14. World Health Organisation: **Global Action Plan for the Prevention and Control of Noncommunicable diseases 2013-2020,**. In. Geneva: World Helth Organisation, ; 2013.
15. Davies M, Macdowall W: **Health promotion theory**. Berkshire, UK,: Open University Press,; 2006.
16. Hyseni L, Atkinson M, Bromley H, Orton L, Lloyd-Williams F, McGill R, Capewell S: **The effects of policy actions to improve population dietary patterns and prevent diet-related non-communicable diseases: scoping review**. *Eur J Clin Nutr* 2017, **71**(6):694-711.
17. World Health Organisation: **Global Status Report on Noncommunicable Diseases 2014**. In. Genva; 2014.
18. Phulkerd S, Lawrence M, Vandevijvere S, Sacks G, Worsley A, Tangcharoensathien V: **A review of methods and tools to assess the implementation of government policies to create healthy food environments for preventing obesity and diet-related non-communicable diseases**. *Implementation science : IS* 2016, **11**:15.
19. Davies S: **Annual Report of the Chief Medical Officer, 2014, The Health of the 51%: Women.** In. London, UK; 2015.

For peer review only - http://bmjopen.bmj.com/site/about/guidelines.xhtml

20. Southampton City Council: **Index of Multiple Deprivation 2015: An analysis of changes since 2010**. In. Southampton 2016.
21. Mortimore A: **Public Health Annual Report 2015**. In. Edited by Coates B, King D. Southampton; 2016.
22. Statacorp: **Stata Statistical Software: Release 13**. In., 11 edn. Texas: College Station; 2013.
23. Vandevijvere S, Swinburn B, International Network for F, Obesity/non-communicable diseases Research M, Action S: **Pilot test of the Healthy Food Environment Policy Index (Food-EPI) to increase government actions for creating healthy food environments**. *BMJ Open* 2015, **5**(1):e006194.
24. Phulkerd S, Vandevijvere S, Lawrence M, Tangcharoensathien V, Sacks G: **Level of implementation of best practice policies for creating healthy food environments: assessment by state and non-state actors in Thailand**. *Public Health Nutr* 2017, **20**(3):381-390.
25. The Food Foundation: **Food Environment Policy Index (Food-Epi) for England**. In. London; 2016.
26. Reynolds CM, Gray C, Li M, Segovia SA, Vickers MH: **Early Life Nutrition and Energy Balance Disorders in Offspring in Later Life**. *Nutrients* 2015, **7**(9):8090-8111.
27. Carletti C, Pani P, Monasta L, Knowles A, Cattaneo A: **Introduction of Complementary Foods in a Cohort of Infants in Northeast Italy: Do Parents Comply with WHO Recommendations?** *Nutrients* 2017, **9**(1).
28. White JM, Begin F, Kumapley R, Murray C, Krasevec J: **Complementary feeding practices: Current global and regional estimates**. *Matern Child Nutr* 2017, **13 Suppl 2**.
29. Summerbell C, Moore H, O'Malley C: **Consequences and determinants of poor nutrition in children aged 0-3 years, and public health interventions that may improve dietary intake: a general review**. *Journal of Children's Services* 2014, **9**(2):128-142.
30. Baillie E, Bjarnholt C, Gruber M, Hughes R: **A capacity-building conceptual framework for public health nutrition practice**. *Public Health Nutrition* 2009, **12**(8):1031-1038.
31. Gonzalez-Zapata LI, Ortiz-Moncada R, Alvarez-Dardet C: **Mapping public policy options responding to obesity: the case of Spain**. *Obes Rev* 2007, **8 Suppl 2**:99-108.
32. Kelly MP, Barker M: **Why is changing health-related behaviour so difficult?** *Public Health* 2016, **136**:109-116.
33. Beauchamp A, Backholer K, Magliano D, Peeters A: **The effect of obesity prevention interventions according to socioeconomic position: a systematic review**. *Obes Rev* 2014, **15**(7):541-554.
34. Lorenc T, Petticrew M, Welch V, Tugwell P: **What types of interventions generate inequalities? Evidence from systematic reviews**. *Journal of Epidemiology & Community Health* 2013, **67**(2):190-193.
35. Middleton J: **Time to put health at the heart of all policy making**. *BMJ* 2017, **357**:j2676.
36. Town and Country Planning Association: **Building the foundations: Tackling obesity through planning and development**. In. London; 2016.
37. Moore MH: **Creating public value : strategic management in government**. Cambridge, Mass.: Harvard University Press; 1995.

For peer review only

38. Roberto CA, Swinburn B, Hawkes C, Huang TT, Costa SA, Ashe M, Zwicker L, Cawley JH, Brownell KD: **Patchy progress on obesity prevention: emerging examples, entrenched barriers, and new thinking**. *Lancet* 2015, **385**(9985):2400-2409.
39. Lachat C, Otchere S, Roberfroid D, Abdulai A, Seret FM, Milesevic J, Xuereb G, Candeias V, Kolsteren P: **Diet and physical activity for the prevention of noncommunicable diseases in low- and middle-income countries: a systematic policy review**. *PLoS Med* 2013, **10**(6):e1001465.
40. Vandevijvere S, Mackay S, Swinburn B: **Benchmarking Food Environments: progress by the New Zealand government on implementing recommended food environment policies and prioritised recommendations**. In. Auckland 2017.
41. Wright CM, Waterston AJR: **Relationships between paediatricians and infant formula milk companies**. *Archives of Disease in Childhood* 2006, **91**(5):383-385.
42. Baird J, Jarman M, Lawrence W, Black C, Davies J, Tinati T, Begum R, Mortimore A, Robinson S, Margetts B *et al*: **The effect of a behaviour change intervention on the diets and physical activity levels of women attending Sure Start Children's Centres: results from a complex public health intervention**. *BMJ Open* 2014, **4**(7):e005290.
43. Lawrence W, Black C, Tinati T, Cradock S, Begum R, Jarman M, Pease A, Margetts B, Davies J, Inskip H *et al*: **'Making every contact count': Evaluation of the impact of an intervention to train health and social care practitioners in skills to support health behaviour change**. *J Health Psychol* 2016, **21**(2):138-151.
44. Yeatman H: **Action or inaction? Food and nutrition in Australian local governments**. *Public Health Nutr* 2009, **12**(9):1399-1407.
45. Middleton G, Henderson H, Evans D: **Implementing a community-based obesity prevention programme: experiences of stakeholders in the north east of England**. *Health Promot Int* 2014, **29**(2):201-211.
46. Sacks G, Swinburn BA, Cameron AJ, Ruskin G: **How food companies influence evidence and opinion – straight from the horse's mouth**. *Critical Public Health* 2017:1-4.
47. Newton A, Lloyd-Williams F, Bromley H, Capewell S: **Food for thought? Potential conflicts of interest in academic experts advising government and charities on dietary policies**. *BMC Public Health* 2016, **16**(1):735.
48. World Health Organisation: **Addressing and managing conflicts of interest in the planning and delivery of nutrition programmes at country level**. In. Geneva, Switzerland; 2016.
49. Piggin J, Hart L: **Physical activity advocacy in the UK: a multiple streams analysis of a hybrid policy issue**. *Leisure Stud* 2017, **36**(5):708-720.

For peer review only

List of figure legends

Figure 1: The schematic diagram of the policy identification process

Figure 2: Percentage of all 57 national policy documents for which each policy option in the policy appraisal framework was present

Figure 3: Percentage of the 8 acts and codes, 21 white papers and 25 evidence-based guidance documents for which each policy option in the policy appraisal framework was present

Figure 4: Percentage of the 10 local policy documents for which each policy option in the policy appraisal framework was present

For peer review only

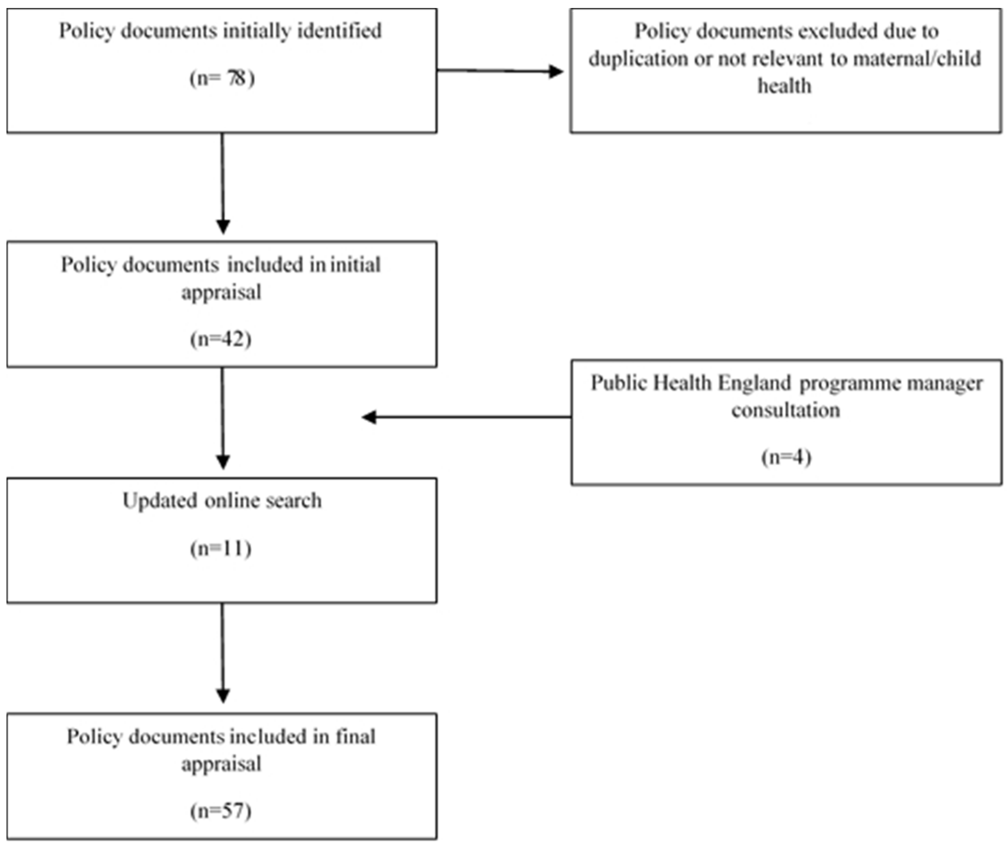

Figure 1: The schematic diagram of the policy identification process

w only

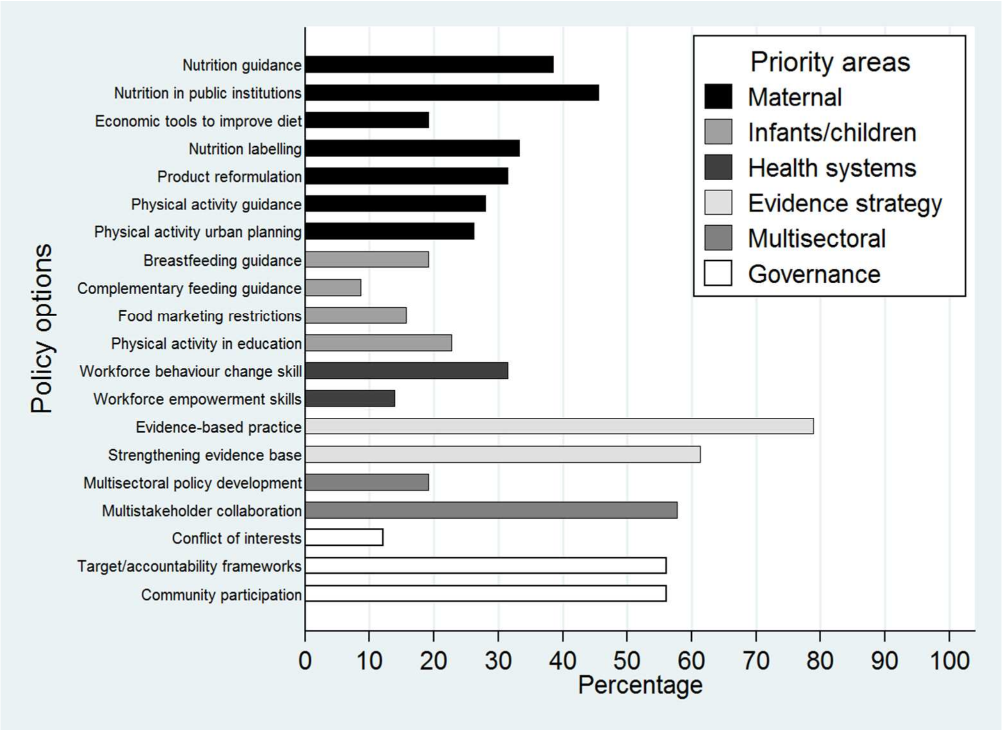

Figure 2: Percentage of all 57 national policy documents for which each policy option in the policy appraisal framework was present

458x333mm (72 x 72 DPI)

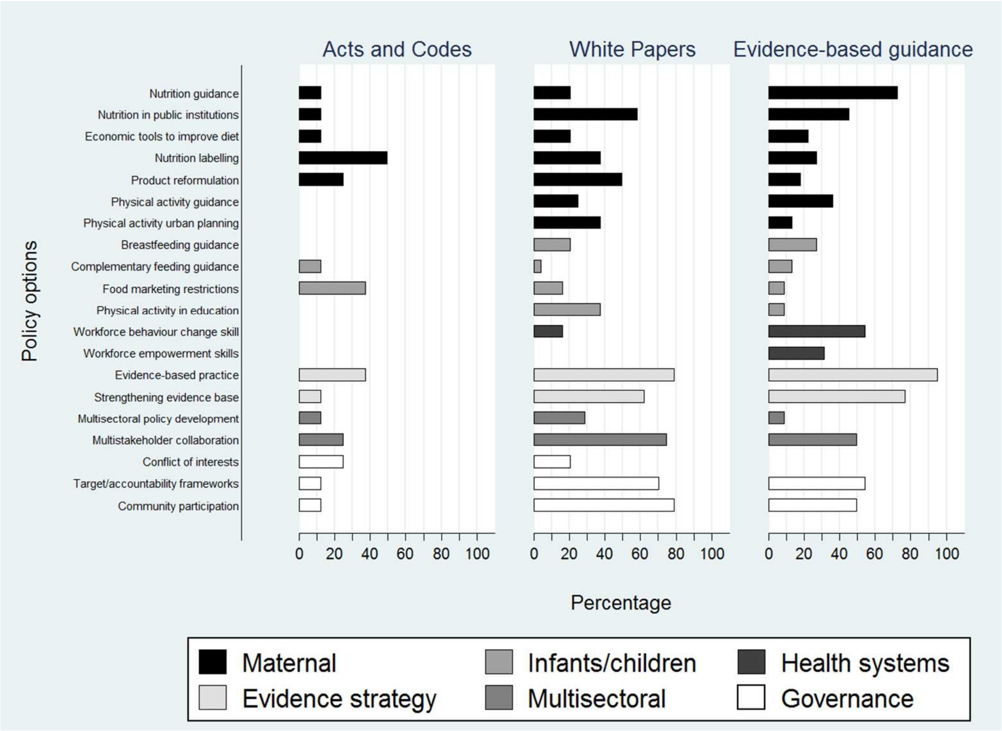

Figure 3: Percentage of the 8 acts and codes, 21 white papers and 25 evidence-based guidance documents for which each policy option in the policy appraisal framework was present

458x333mm (72 x 72 DPI)

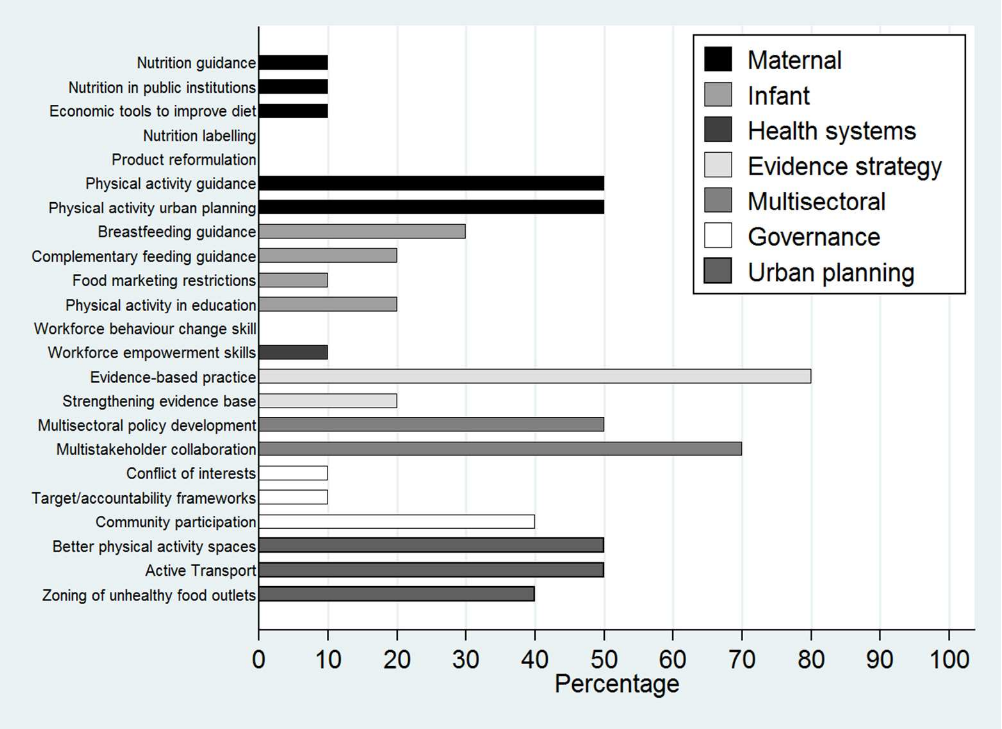

Figure 4: Percentage of the 10 local policy documents for which each policy option in the policy appraisal framework was present

458x333mm (72 x 72 DPI)

Additional material

Appendix 1: The Policy Appraisal Framework used to analyse the policy documents identified

Priority Areas	Policy options	Detail	Key words for data extraction	Inclusion [a,b]
**Maternal health**<br>*Objective 3: Reducing modifiable risk factors*<br>*Overarching principle : life-course approach*	1. Social media campaigns and/or dietary guidance	39.8	social media, nutrition, education, healthy eating, health promotion, social marketing, diet, food, guidelines, guidance, healthy lifestyle, mass media, campaigns, health promotion, eat well plate, 5 a day.	Inclusion: Makes reference to dietary education and/or materials including reference to dietary guidelines or intent to develop dietary guidelines. Includes material regarding adolescent females. Can include reference to external guidance linked to in the policy document that meet the criteria, as long as the external guidance is accurately referenced in the correct corresponding place in the policy document.<br>Exclusion: Education initiative solely focusing on children's diets, breastfeeding (except diets for breastfeeding women), alcohol consumption and smoking.
	2. Healthy food and health promotion in public institutions	39.5, 39.9, 41.4 (iv)	public institute, standard, guideline, healthy environment, active transport, government agencies, food, nutrition, physical activity exercise, movement, school, hospital, meals.	Inclusion: Reference to details /guidelines/health promotion of food, diet and physical activity health promoting environments within public institution. Includes reference to schools, hospitals and any government agencies.<br>Exclusion: smoke-free, alcohol and breastfeeding environments
	3. Economic tools to increase healthy and reduce less healthy food consumption	39.6<br>30.2	taxes, subsidies, sugar sweetened beverages, fiscal strategies, fruit and vegetable, finance, vouchers.	Inclusion: Must make reference to intent to increasing consumption of healthy foods and reducing consumption of less healthy foods.<br>Exclusion: Physical activity or public transport subsidies

For peer review only - http://bmjopen.bmj.com/site/about/guidelines.xhtml

	4. Nutrition labelling	39.10	food labelling, nutrition labelling, packaged food, front of pack, back of pack, traffic lights, health claims, nutrition information panel	Inclusion: Must make reference to nutrition information or improving health. Labelling on pre-packaged foods or nutrition facts or content about foods provided by an establishment. For example hospital catering.<br>Nutrition labelling for infants and babies including baby formula is included.<br>Exclusion: Reference to food safety.
	5. Guidelines to reduce sodium, saturated fat, free sugar and increase fruit and vegetables for manufacturers to create healthy agriculture, retail and catering sectors	39.3, 39.4, 39.7	sodium, salt, trans (fat) saturated fat, (free) sugars, portion/serve size, fruit and vegetables, reformulation. Agriculture, farming, food retailers, caterers, subsidies, land availability, energy density.	Inclusion: Product reformulation and the improvement of the nutritional quality of the food – specifically saturated/trans fat, sugars, salt, fruit and vegetables, energy density or portion sizes. Not required to directly refer to maternal health. Includes agricultural, manufacturing, retail and catering sectors. Includes reference to changing purchasing of a product with less salt, sodium, sugar, or fat of catering purposes etc.
	6. Physical activity guidelines and/or public campaigns	41.4 (iii), 41.4 (v), 41.5, 41.1	mass media, physical activity, inactivity, sedentary, active transport, exercise, healthy lifestyle, mass media, social media, leaflet, booklets,	Inclusion: Reference to guidelines/recommendations for physical activity, exercise and active transport or ways to reduce inactivity or intent to develop physical activity or related guidelines Includes material regarding adolescent females.<br>Exclusion: Education initiatives solely focusing on children.

For peer review only - http://bmjopen.bmj.com/site/about/guidelines.xhtml

	7. Urban, transport and recreation planning to increase daily activity	41.4 (i), 41.4 (iv)	urban planning, urban transport, recreation, infrastructure walking, cycling, safety, active transport, green space, natural environments, lockers, facilities, shower.	Inclusion: Physical activity and active transport facilities accessibility, availability, affordability and safety. Includes community playgrounds and outdoor spaces. Is about the encouragement forms of active transport leading to improved physical activity.<br>Exclusion: Initiatives to enhance social connectivity.
Urban Planning [c]	8. Green Spaces and physical activity facilities	41.4 (iv)	green Spaces, playgrounds, community gyms, physical activity, sports, parks	Inclusion: Increase number and improvement of community green spaces and the availability of council physical activity facilities that enable active options
	9. Active Transport	41.4<br>41.4 (i)	traffic safety, walking cycling, footpath, pathway, active transport	Inclusion: Safety in terms of traffic lights and street lights and safety to enable active transport including footpaths and any sort of pathway and availability of community bikes. Is about direct changes at a local policy level that improve the opportunity for active transport. This includes changes to infrastructure and physical environmental improvements.
	10. Licensing or Zoning of less healthy food outlets	39.7<br>39.4	zoning, takeaway outlets, diary, corner store	Inclusion: Licensing and zoning to regulate the density of less healthy food outlets
**Infant health**<br><br>*Objective 3: Reducing modifiable risk factors Overarching principle : life-course approach*	1. Breastfeeding guidelines/ initiatives	39.1, 18.5, Annex 43(i)	breastfeed, nursing	Inclusion: Recommendations for breastfeeding and reference made to breastfeeding recommendations or action plan. Includes promotion of breastfeeding friendly environments and encouragement or promotion of breastfeeding. Reference to breastfeeding guidelines. Includes link to external sources with explicit mention of the criteria.<br>Exclusion: Food and dietary guidelines for breastfeeding women.

For peer review only - http://bmjopen.bmj.com/site/about/guidelines.xhtml

	2. Complementary feeding guidelines/ initiatives	39.1	complementary feeding, introducing solids, weaning	Inclusion: Reference to recommendations/ guidelines for complementary feeding and/or introducing solids, including infant and toddlers dietary guidelines.
	3. Guidelines/ restrictions for the marketing of food and beverages to children	33, 38, 39.2, 43.1	marketing, advertising, promotions, sponsorship	Inclusion: Details/restrictions of food marketing or advertising to children or reference made to food marketing/advertising guidelines. Includes television, online, sponsorship of sports, school and other recreation activities. Also included is marketing for children, not necessarily targeted at children but is targeted to them, for example baby formula marketing.
	4. Physical activity opportunities in education institutions	41.4 (ii)	physical education, active movement, walking , cycling, physical activity, exercise, active transport, preschools, day care, Kindergartens, child care, outdoor play	Inclusion: Reference made to guidelines/ recommendations to physical activity options for education institutions. Includes requirements for making changes to environment to encourage physical activity. Includes initiatives external to school grounds but school-driven such as 'walking school bus'. Needs to refer to education institutions including pre-schools to universities. Exclusion: Initiatives solely focusing on staff of these institutions.
**Strengthening health systems** *Objective 4 Overarching principle: Empowerment of people and*	1. Improving the knowledge, skills and motivation of the workforce (maternal and infant health professionals) through training in NCD and obesity prevention, specifically regarding behaviour change, diet and physical activity	48.4 (i, ii), 48.3(vii 30.8	skills, development, education, workforce develop, knowledge, training, behaviours skills	Inclusion: Regards training of health professionals inclusive of Maternal and Infant health professionals to increase knowledge and skills in behaviour change, diet and/or physical activity requirements, inclusive of mothers (preconception, pregnancy and breastfeeding) and infants. Is about NCD prevention. Exclusion: Not including smoking or alcohol.

For peer review only - http://bmjopen.bmj.com/site/about/guidelines.xhtml

communities	2. Maternal and Infant health professionals empower people to manage their own condition and support family-centred/self-care, particularly among high-risk populations.	48.3 (vi),, 49.4 (iii)	empower, Patient/family/ community centre, self-care, cultural competent, cultural awareness, minority groups, low-income groups.	Inclusion: Reference to training of health professionals inclusive of Maternal and Infant health professionals in skills that ensure the level of care is patient/family centred and/or empowering or a particular consideration for high risk populations. Not just about training but also competencies and training to adequate levels to provide empowerment. Includes empowerment of people who help target group, e.g. carers, parents, etc.<br>Exclusion: Blanket open statements about person centred approaches.
**Evidenced based strategies**<br>*Overarching principle*	1. Adoption of evidence based practice	18.7<br>32.1<br>39.8<br>41.5	best practice, evidence, strategies, evidence informed/based	Inclusion: Must refer to the use of evidence-base strategies (latest scientific evidence and/or best practice) within the development and implementation of policies/programmes /interventions.
	2. Strengthen evidence base for effective dietary and physical activity interventions through research and evaluation	53.5<br>41.6	research, evaluation, scientific, knowledge base, best practice	Inclusion: Refers to research and evaluations of interventions/programmes to improve diet and/or physical activity. Includes the documents referring to the need for review or development guidance.
**Multisectoral action**<br>*Objective 2*<br>*Overarching principle*	1. Multisectoral development of the policy	18.4<br>27<br>29<br>30.5<br>30.6	*searched the policy authors and the introduction of the policy.*	Inclusion: Reference to the process of policy development. Must refer to more than one government department/government funded institution in policy development.
	2. Multistakeholder collaboration and cooperation	21.3<br><br>39.3<br>41.3	collaboration, co-operation, engagement, stakeholder, community, local government.	Inclusion: Development of collaborations /engagements with stakeholders for interventions/programme/policies. Includes stakeholders from multiple sectors, such as NGOs, industry, civil society groups, professional bodies, different disciplines, etc.<br>Exclusion: Not required to be directly related to diet/food and physical activity behaviour change environment. The documents can't just include multistakeholder action as a throwaway comment but must name examples.

For peer review only - http://bmjopen.bmj.com/site/about/guidelines.xhtml

**Governance and accountability** *Objective 2 Overarching Principle: Management of real, perceived or potential conflicts of interest*	1. Management or acknowledgement of conflicts of interest	38<br><br>18.9	conflicts of interest, conflicting industries/ bodies, funding sources, guidelines	Inclusion: Reference to the explicit awareness of conflicts of interest with development of diet and physical activity, healthy environments and initiatives<br>Exclusion: Initiatives related to smoking and alcohol consumption.
	2. Set targets/ establish accountability frameworks for NCDs, diet or physical activity behaviours	29<br>30.7<br><br>Pg. 5. Voluntary Global Targets	target, framework, accountability, plan, KPI's, indicators, outcomes, outputs, goal, objective, vision	Inclusion: Must refer to a framework or targets set by WHO or National Governments that relate to NCDs, obesity, diet and physical activity. Includes intention to develop a framework or targets.<br>Exclusion: Accountability frameworks for Acts/policies that do not explicitly relate to NCD prevention i.e. professional code of conduct accountability frameworks.
	3. Implement participatory, community based approaches	 30.10<br>48.1 (ii)	participation, community, empowerment, involvement, capacity building, consultation.	Inclusion: Reference to community members/representative groups involvement in the development/ implementation of interventions/programmes and policy development. Must recognise that the community have a key role to play, not just about guidelines but how the local community are involved.

[a] It is acceptable for links to external documents to be included as a positive result for the different policy options. However the external documents must be linked to in the body of the text of the policy document being appraised and must the body of text must explicitly mention the policy option area being explored.
[b] Focusses on the physical components of health related to physical activity and diet, therefore doesn't include mental health in this instance.
[c] This section only applies to the Local policies from the case study.
[d] The colouring corresponds to the ecological model of health levels: light grey is individual behaviour change, grey is supportive social and physical environments in institutions, and dark grey is macro environmental policy.

For peer review only - http://bmjopen.bmj.com/site/about/guidelines.xhtml

Appendix 2: List of all policy documents appraised arranged by policy type

Policy Document Title	Date Published	Author
Acts and codes		
The Processed Cereal–Based Foods and Baby Foods for Infants and Young Children (England) Regulations	December 2003	Department of Health
The Childcare Act 2006	July 2006	Ministry of Justice
The Infant Formula and Follow-on Formula (England) Regulations	December 2007	Department of Health
The UK Code of Non-Broadcast Advertising, Sales Promotion and Direct Marketing	August 2010	Advertising Standards Authority Ofcom
The UK Code of Broadcast Advertising (BCAP code)	August 2010	Advertising Standards Authority Ofcom
Health and Social Care Act 2012	June 2012	Department of Health
Children and Families Act 2014	March 2014	Department for Education<br>Ministry of Justice<br>Department for Business, Innovation & Skills[a]<br>Department for Work and Pensions<br>Department of Health
The Food Information Regulations 2014	July 2014	Department for Environment, Food and Rural Affairs
White papers		
Healthy Weight Healthy Lives	January 2008	Department of Health<br>Department for Children, Schools and Families[b]
Healthy Lives, Brighter Futures: the strategy for children and young people's health	February 2009	Department of Health<br>Department for Children, Schools and Families[b]
Healthy Lives Healthy People: our strategy for public health in England	November 2010	Department of Health
Health Visitor Implementation Plan 2011-2015: a call to action	February 2011	Department of Health
Supporting Families in the Foundation Years	August 2011	Department for Education<br>Department of Health
Joint Strategic Needs Assessment and Joint Health and Wellbeing Strategies Explained	December 2011	Department of Health
National Planning Policy Framework	March 2012	Department for Communities and Local Government
An Update in the Government's Approach to Tackling Obesity	July 2012	National Audit Office

For peer review only

Improving Children And Young People's Health Outcomes: a system wide response	February 2013	Department of Health
Giving all Children a Healthy Start in life	March 2013	Department for Education<br>Department of Health
Developing a Specification for Lifestyle Weight Management Services	March 2013	Department of Health
National Health Visitor Plan: progress to date and implementation 2013 onwards	June 2013	Department of Health<br>NHS England<br>Public Health England<br>NHS Health Education England
Briefing on the Government's Ambition for Cycling	August 2013	Department for Transport
Moving More, Living More: Olympic and Paralympic Games legacy	February 2014	Cabinet Office<br>Department of Health<br>Department for Digital, Culture, Media and Sport,<br>Department for Education<br>Mayor of London
Maternity and Early Years: making an good start to family life	March 2015	Department of Health
The Governments Responsibility Deal	March 2015	Department of Health
Statutory Guidance on Joint Strategic Needs Assessments and Joint Health Wellbeing Strategies.	March 2015	Department of Health
A Plan for Public Procurement: food & catering - balanced scorecard for public food procurement	July 2015	Department for Environment, Food and Rural Affairs
Five Year Forward View	October 2015	NHS England
Public Health Outcomes Framework, 2013-2016 (1A and 1B)	November 2015	Department of Health
Childhood Obesity: a plan for action	August 2016	Department of Health
Soft Drinks Industry Levy	December 2016	HM Revenue and Customs<br>HM Treasury
Evidence-based guidance documents		
Postnatal Care up to 8 Weeks After Birth	July 2006	NICE
Obesity Prevention	December 2006	NICE
Food Standards Agency Nutrient and Food Based Guidelines for UK Institutions	October 2007	Food Standards Agency
Behaviour Change: general approaches	October 2007	NICE

For peer review only - http://bmjopen.bmj.com/site/about/guidelines.xhtml

Antenatal Care for Uncomplicated Pregnancies	March 2008	NICE
Everybody Active, Every Day: an evidence-based approach to physical activity	October 2014	Public Health England
The Link Between Pupil Health and Wellbeing and Attainment: a briefing for head teachers, governors and staff in education settings	November 2014	Public Health England
Behaviour Change: individual approaches	January 2015	NICE
Physical Activity: for NHS staff, patients, and carers.	March 2015	NICE
Weight Management Before, During and After Pregnancy	July 2015	NICE
Postnatal Care	July 2015	NICE
Maternal and Child Nutrition	July 2015	NICE
A Quick Guide to the Government's Healthy Eating Recommendations	August 2015	Public Health England
Obesity in Children and Young People: prevention and lifestyle weight management programmes	August 2015	NICE
Multiple Pregnancy: twin and triplet pregnancies	September 2015	NICE
Sugar Reduction: the evidence for action	October 2015	Public Health England
From Evidence into Action: opportunities to protect and improve the nation's health	October 2015	Public Health England
Obesity and the Environment: regulating the growth of fast food outlets.	November 2015	Public Health England<br>Local Government Association
Nutrition Support in Adults	November 2015	NICE
The Influence of Maternal, Foetal and Child Nutrition on the Development of Chronic Disease in Later Life	November 2015	Scientific Advisory Committee on Nutrition
Obesity in Adults: pre obesity in adults: prevention and lifestyle weight management lifestyle weight management programmes	January 2016	NICE
Building the Foundations: tackling obesity through planning and development	February 2016	Local Government Association<br>Town and Country Planning Association<br>Public Health England
Healthier Food Procurement	August 2016	Local Government Association

For peer review only

Antenatal Care	September 2016	NICE
Childhood Obesity: brave and bold action	November 2016	House of Commons Health Committee
Guide to Creating a Front of Pack (FoP) Nutrition Label for Pre-packed Products Sold Through Retail Outlets	November 2016	Department of Health<br>Food Standards Agency<br>Welsh Government<br>Northern Ireland Government<br>Food Standards Scotland<br>British Retail Consortium
Healthy Weight, Healthy Futures: local government action to tackle childhood obesity	February 2017	Local Government Association
Local Policies		
Local Transport Plan 3: strategy and implementation plan for Southampton.	March 2011	Southampton City Council
Supporting Carers in Southampton: a joint strategy for improvement 2011-2015	December 2012	Southampton City Council<br>Southampton City Clinical Commissioning Group
Southampton's Joint Health and Wellbeing Strategy: gaining healthier lives in a healthier city	January 2013	Southampton City Council<br>Southampton City Clinical Commissioning Group
Southampton Local Plan: compliance with national planning policy framework	June 2013	Southampton City Council
Southampton City Strategy 2014-2025	July 2014	Southampton City Council
Southampton Safe City Strategy 2014-17	July 2014	Safe City Partnership (Hampshire Constabulary, Southampton City Council and other representatives)
Our Vision for Future Southampton	June 2015	Southampton City Council
Health and Wellbeing Strategy 2017-2025	March 2017	Southampton City Council<br>Southampton City Clinical Commissioning Group
Cycling Southampton: a cycling strategy for our city 2017-2027	June 2017	Southampton City Council
Children and Young People's Healthy Weight Plan	June 2017	Southampton City Council

[a] superseded by Department for Business, Energy and Industrial Strategy, [b] superseded by Department of Education

For peer review only

BMJ Open

How well do national and local policies in England relevant to maternal and child health meet the international standard for non-communicable disease prevention? A policy analysis

Journal:	*BMJ Open*
Manuscript ID	bmjopen-2018-022062.R1
Article Type:	Research
Date Submitted by the Author:	05-Jul-2018
Complete List of Authors:	Penn-Newman, Daniel; University of Southampton, MRC Lifecourse Epidemiology Unit<br>Shaw, Sarah; University of Southampton, MRC Lifecourse Epidemiology Unit<br>Congalton, Donna; University of Auckland, Liggins Institute<br>Strommer, Sofia; University of Southampton, MRC Lifecourse Epidemiology Unit<br>Rose, Taylor; University of Southampton, MRC Lifecourse Epidemiology Unit<br>Lawrence, Wendy; University of Southampton, MRC Lifecourse Epidemiology Unit<br>Chase, Debbie; Southampton City Council, Public Health<br>Cooper, Cyrus; University of Southampton, MRC Lifecourse Epidemiology Unit; NIHR Southampton Biomedical Research Centre, University Hospital Southampton NHS Foundation Trust<br>Barker, Mary; University of Southampton, MRC Lifecourse Epidemiology Unit; NIHR Southampton Biomedical Research Centre, University Hospital Southampton NHS Foundation Trust<br>Baird, Janis; University of Southampton, MRC Lifecourse Epidemiology Unit<br>Inskip, Hazel; University of Southampton, MRC Lifecourse Epidemiology Unit; NIHR Southampton Biomedical Research Centre, University Hospital Southampton NHS Foundation Trust<br>Vogel, C; University of Southampton, MRC Lifecourse Epidemiology Unit
**Primary Subject Heading**:	Public health
Secondary Subject Heading:	Health policy
Keywords:	Health policy < HEALTH SERVICES ADMINISTRATION & MANAGEMENT, maternal health, child health, Nutrition < TROPICAL MEDICINE, physical activity, PUBLIC HEALTH

SCHOLARONE™
Manuscripts

For peer review only

How well do national and local policies in England relevant to maternal and child health meet the international standard for non-communicable disease prevention? A policy analysis

Daniel Penn-Newman[1], Sarah Shaw[1], Donna Congalton[2], Sofia Strommer[1], Taylor Rose[1], Wendy Lawrence[1,3], Debbie Chase[4], Cyrus Cooper[1,3], Mary Barker[1,3], Janis Baird[1], Hazel Inskip[1,3], Christina Vogel[1]

[1] Medical Research Council Lifecourse Epidemiology Unit, University of Southampton, Southampton General Hospital, Tremona Road, Southampton, SO16 6YD, UK

[2] Liggins Institute, University of Auckland, Level 2, Building 505, 85 Park Rd, Grafton, Auckland 1142, NZ

[3] NIHR Southampton Biomedical Research Centre, University Hospital Southampton NHS Foundation Trust and University of Southampton University Hospital Southampton NHS Foundation Trust and University of Southampton, Southampton SO16 6YD, UK

[4] Public Health, Southampton City Council, Civic Centre, Southampton SO14 7LY, UK

Correspondence to: Christina Vogel, cv@mrc.soton.ac.uk, Tel: 023 8076 4042, University of Southampton, Southampton General Hospital, Tremona Road, Southampton SO16 6YD, UK

Word count: abstract: 291, main text: 5233

For peer review only

Abstract

Objectives: i) To identify national policies for England and local policies for Southampton City that are relevant to maternal and child health. ii) To quantify the extent to which these policies meet the international standards for nutrition and physical activity initiatives set out in the World Health Organisation Global Action Plan for the Prevention and Control of Non-Communicable Diseases (NCDs) (WHO Action Plan).

Design: The policy appraisal process involved three steps: i) identifying policy documents relevant to maternal and infant health from , ii) developing a policy appraisal framework from the WHO Action Plan, and iii) analysing the policies using the framework.

Setting: England and Southampton City

Participants: 57 national and 10 local policies

Results: Across both national and local policies, priority areas supporting public health processes, such as evidence-based practice, were adopted more frequently than the action-orientated areas targeting maternal and child dietary and physical activity behaviours. However, the policy option managing conflicts of interest was rarely considered in the national policies (12%), particularly in white papers or evidence-based guidelines. For the action-orientated priority areas, maternal health policy options were more frequently considered than those related to child health or strengthening health systems. Complementary feeding guidance (9%) and workforce training in empowerment skills (14%) were the least frequently action-orientated policy options adopted among the national policies. The maternal nutrition-focused and workforce development policy options were least frequent among local policies adopted in 10% or fewer. Macro-environmental policy options tended to have a lower priority than organisational or individual options among national policies (p=0.1) but had higher priority among local policies (p=0.02).

For peer review only

Conclusions: Further action is needed to manage conflicts of interest and adopt policy options that promote a system-wide approach to challenging NCDs caused by poor diet and physical inactivity.

Key words: policy, maternal health, child health, nutrition, physical activity, public health

Strengths and limitations of this study

- This is the first polity review to focus on maternal, infant and child health for non-communicable disease prevention and considers government activity at both national and local levels
- The assessment method reflects adherence to the World Health Organisation international best practice and enable easy identification of areas for improvement
- Use of a second assessor and verification by policy makers provided quality assurance
- Policies from only one local authority were assessed as a case study
- The use of a dichotomous measure does not provide comprehensive details about the scale of policy implementation

1. Introduction

Noncommunicable diseases (NCDs) including cardiovascular disease and diabetes are the primary cause of death globally.[1] In the United Kingdom (UK), they account for 89% of all adult deaths.[2] Exposure to risk factors such as obesity, physical inactivity and poor diet begins early, before conception and in utero.[3] Accordingly, optimising maternal, infant and child health is a top priority on the international health agenda. There is thus a need to align the NCD prevention agenda with the high level directives encouraging member states to enact nutrition initiatives targeting preconception, pregnant and breastfeeding mothers, and the first 1000 days.[4-6]

For peer review only - http://bmjopen.bmj.com/site/about/guidelines.xhtml

National and local governments' policies play a key role in leading action on NCD prevention. National governments are best placed to monitor population behaviours and health indicators, and have a responsibility to enact policies that will optimise the health of their citizens.[7] Local governments have the ability to work with the community, respond to local needs and create local settings that support healthier lifestyle behaviours, particularly in the UK where public health responsibilities were deferred to local authorities in 2012.[8] The content of local and national policies may vary considerably or may be closely aligned, depending on whether there is strong national leadership on an issue or high heterogeneity in local authorities' structures and priorities.[9] While jurisdictional responsibilities differ between government levels and only national governments can enact legislation, for example on nutrition labelling, there are activities that local governments can adopt on the same issue, such as shelf or menu nutrition prompts in cafeterias in schools, hospitals or leisure centres.

National government policies cover a variety of document types that hold different levels of authority from clear government directive to recommending an action in a normative sense. One set of categories applicable in the UK includes: i) acts and codes - legally binding approved by the House of Commons and Lords that are implemented by an appropriate government agency [10], ii) white papers – an outline of government's strategic direction or priorities for action on a particular subject [11], and iii) evidence-based guidelines – implicit policies including summaries of evidence into advice on best-practice standards or recommended course of action, such as guidance by the National Institute for Health and Care Excellence (NICE).[12] Different types of national policies may vary in scope or represent comprehensive, complementary action depending on a government's political ideology, time in office and power across the parliamentary houses, amongst other factors.[13] Policy options to address an issue are often raised initially in policy documents with less authority, to be

For peer review only

discussed and debated in parliament before adoption as a government directive. Assessing similarities across or differences between types of policy documents can provide insight into areas for improvement, particularly when compared to international standards.

To strengthen state members' efforts to address the growing global burden of NCDs, the 66th World Health Assembly endorsed the World Health Organisation (WHO) Global Action Plan for the Prevention and Control of NCDs (Action Plan) and NCD Global Monitoring Framework in 2013.[14] The Action Plan recognises the primary role of governments in responding to the growing challenge of NCDs and highlights the links between poor-diet, physical inactivity and NCDs. It also states the importance of enhancing maternal and child health. The Action Plan sets out a range of policy options that governments at all levels can adopt and provides an international standard against which government action can be assessed. The policy options work across the ecological model of health. [15] They target: i) individual-level behaviour change, ii) organisational-level determinants including social and physical settings, and iii) macro-environmental-level determinants including fiscal policy and infrastructure. Multi-component policy action that works across these levels has shown to be more effective at improving lifestyle behaviours than action at a single level,[16] though it is recognised that macro-environmental policy action is politically challenging.[7]

The current NCD Global Monitoring Framework measures progress in reducing prevalence rates of health outcomes or risk factors but does not comprehensively assess progress on policy options to improve both dietary and physical activity behaviours.[17] Other policy appraisal frameworks focus on national government progress for a single behaviour and have not considered policy options specific to maternal, infant and child health.[13 18] The 2014 report of the Chief Medical Officer highlighted that half the population of women aged 25-34

For peer review only - http://bmjopen.bmj.com/site/about/guidelines.xhtml

years in England are overweight or obese and noted the need to improve dietary and physical activity behaviours among women of reproductive age.[19] Additionally, the public health white paper for England 'Healthy Lives Healthy People' and the 'Childhood Obesity' strategy highlight the need for action across government sectors and levels to address the persistent public health issue of obesity. Assessment of how well national and local government policies are supporting women and children to adopt healthy dietary and physical activity behaviours is needed to identify gaps for further government action. Therefore, this study aimed to quantify the extent to which English national and local government policies that are relevant to maternal and child health meet the international standard set out in the WHO Action Plan. There were three aims of this study. First, to identify national English government policies and local government policies, using Southampton City as a case study. Second, to analyse the extent of adoption of the Action Plan policy options in these policies. Third, to assess differences in adoption of the policy options by policy type (local/national and acts/white papers/evidence-based guidance), and across three levels of an ecological model of health.

3. Methodology

The policy appraisal process involved three steps. The first step involved identification of national (England) and local policies (Southampton City) that could influence maternal and child health. The second step involved developing a policy appraisal framework based on relevant WHO Action Plan policy options. The third step was to analyse the identified policies using the appraisal framework. Ethical approval was not required for this study because it was a secondary analysis of publicly available policy documents.

3.1 Identification of National and Local Policy Documents

For peer review only - http://bmjopen.bmj.com/site/about/guidelines.xhtml

The policies were identified in December 2015 and an updated search was conducted in June 2017. At each time point, government websites were exhaustively searched to identify acts and codes, briefing notes, white papers and evidence-based guidelines that were relevant to maternal and child health. We adopted a broad definition of maternal and child health policy by including policy documents that targeted the health of women and/or children, as well as those that could indirectly influence the health or dietary or physical activity behaviours of women and children. Accordingly, a range of websites were searched including the Department of Health, Department for Education, Department for Children Schools and Families, and the Department for Communities and Local Government. Additionally, executive agency websites, including the Food Standards Agency, Public Health England, NICE and the Local Government Association, were searched because these agencies can influence government policy development. Policies focusing on disease treatment or specific nutrients, or those detailed in policies published at a later date were excluded. The compilation of the identified national policies was then sent to a programme manager at Public Health England for verification and identification of missing policies. Documents published during the UK Coalition and Conservative Governments, from May 2010 to June 2017, were the focus of this review. Key policies prior to this date were included if they: i) informed current policies, or ii) had not been superseded.

Southampton City was used as a case study for the local policy analysis. Southampton is a large city on the south coast, more deprived than other cities in the affluent South East of England. Southampton is ranked the 67th most deprived of the 326 Local Authorities in England.[20] In 2014/15, 47% of women in Southampton were classified as overweight or obese at their maternity booking appointment,[21] indicating that this local health issue is similar to the national and international level. To aid methodological rigor and completeness, the identification of local Southampton policies was supported and verified by a public health

For peer review only

consultant (DChase) at the Southampton City Council. Policies were extracted from the Southampton City Council website or provided by City Council staff.

3.2 Development of a Policy Appraisal Framework

Three researchers (CV, DCongalton, DPN) independently identified components of the WHO Action Plan relevant to maternal and child health, and dietary or physical activity behaviours that could be categorised into priority areas for the appraisal framework. Each researcher then highlighted policy options from the WHO Action Plan that were relevant for maternal (preconception, pregnancy, lactation and motherhood) and/or infant and child health. The policy options were specific initiatives that could be identified in policies. All policy options were independent from each other and corresponded with a single priority area. Additional material Appendix 1 presents the final appraisal framework agreed upon by the three researchers. Discrepancies were resolved by returning to the WHO Action Plan to ensure consistency and accuracy.

A total of six priority areas were established for the appraisal framework. Three priority areas were action-orientated: i) maternal health, ii) infant/child health, iii) strengthening health systems. The remaining three represent public health processes necessary for high-quality policy development: iv) evidence-based strategies, v) multisectoral action, and vi) governance and accountability. Each priority area had between two and seven accompanying policy options (see Additional material, Appendix 1 for details). A total of 20 policy options were included in the national policy appraisal framework. The same 20 were included in the local policy appraisal framework because policy options such as fiscal, labelling, marketing or reformulation initiatives could also be employed in local settings such as schools, leisure centres or hospitals through local government commissioning contracts or guidelines. Three additional urban planning policy options were also included to encompass local government

For peer review only - http://bmjopen.bmj.com/site/about/guidelines.xhtml

jurisdiction over urban planning. To ensure consistency in identifying the presence or absence of a policy option, search terms and inclusion and exclusion criteria for each policy option were created (Additional material, Appendix 1).

The policy options were categorised into three levels in line with the ecological model of health: individual, organisational and environmental (Additional material, Appendix 1). Seven policy options could not be categorised according to the three levels of the ecological model because they were not action-orientated but represented good public health processes.

3.3 Policy Appraisal Process

One researcher (DPN) assessed all identified policies against the appraisal framework. Policies were reviewed individually and search terms (Additional material, Appendix 1) were used to pinpoint relevant sections that were comprehensively assessed to determine whether or not the policy option was present. The inclusion and exclusion criteria for each policy option were used to guide this procedure. Random selections, comprising 44% of the national policies and 50% of the local policies, were double coded by a second researcher (SShaw). Inconsistencies in coding were found in 4% of the double coded policies, indicating over 95% level of agreement. Discrepancies were discussed with a third researcher (CV) and agreement was reached after returning to the policy and appraisal framework.

3.4 Statistical analysis

Descriptive analyses were used to describe the policies by type, author, and date of publication. The frequency for each of the policy options was calculated to ascertain their representation across the national and the local policies. Frequencies of policy options were also calculated according to the three national policy types: acts and codes, white papers and evidence-based guidelines. Cochran’s Q test was used to test for differences in policy option

For peer review only - http://bmjopen.bmj.com/site/about/guidelines.xhtml

frequency across the three ecological levels (individual, organisational, and environmental) to identify whether there was difference in policy option adoption according to level of action. All statistical analyses were conducted using Stata statistical software package version 13.[22]

3.5 Patient and public involvement

The public was not involved in this study because it was a policy document analysis that did not involve participants.

4. Results

4.1 Characteristics of Identified Policies

A total of 57 national policies relevant to maternal and child health were identified; 47 were published between May 2010 and June 2017, and 10 prior to 2010. Figure 1 provides a schematic representation of the national policy identification process.

Evidence-based guidance documents (n=27, 47%) and white papers (n=22, 39%) represented the vast majority of identified policies, with acts and codes representing the remainder (n=8, 14%). Additional material Appendix 2 details the title, publication date and authors of each identified policy according to the three policy types. Of the 28 organisations that authored the national policies, the Department of Health was most prolific, authoring more than one third of all policies (n=21, 37%; Table 1), including half of the acts (n=4, 50%) and almost three quarters of the white papers (n=16, 73%). NICE was the second most common author (n=14, 25%), publishing the majority of the evidence-based guidance documents (n=14, 52%). Most policies were authored by a single organisation (n=46, 81%). However, 14 organisations were

For peer review only

co-authors. The Department of Health was the most common co-author (8 of the 11 co-authored policies).

Table 1: List of authors for identified national policies

Author	Number of single authored documents	Number of co-authored documents	Total[a] n (%)
Department of Health	13	8	21 (37)
National Institute for Health Care Excellence	14	0	14 (25)
Public Health England	5	3	8 (14)
Department of Education[b]	0	6	6 (11)
Local Government Association	2	2	4 (7)
Advertising Standards Authority/ Ofcom	2	0	2 (4)
Department for Communities and Local Government	1	1	2 (4)
Department for the Environment, Food and Rural Affairs	2	0	2 (4)
Food Standards Agency	1	1	2 (4)
Ministry of Justice	1	1	2 (4)
National Health Service (NHS) England	1	1	2 (4)
British Retail Consortium	0	1	1 (2)
Cabinet Office	0	1	1 (2)
Department for Business, Innovation and Skills[c]	0	1	1 (2)
Department for Digital, Culture, Media and Sport	0	1	1 (2)
Department for Work and Pensions	0	1	1 (2)
Department of Transport	1	0	1 (2)
Food Standards Scotland	0	1	1 (2)
HM Revenue and Customs	0	1	1 (2)
HM Treasury	0	1	1 (2)
House of Commons Health Committee	1	0	1 (2)
Mayor of London	0	1	1 (2)
National Audit Office	1	0	1 (2)
NHS Health Education England	0	1	1 (2)
Northern Ireland Government	0	1	1 (2)

For peer review only

Scientific Advisory Committee on Nutrition	1	0	1 (2)
Town and Country Planning Association	0	1	1 (2)
Welsh Government	0	1	1 (2)

[a] column total reflects the total number of national policy documents published by each author and rounded percentage of all national policies (n=57) [b] Department for Children, Schools and Families superseded by Department of Education, [c] Department for Business, Innovation and Skills superseded by Department for Business, Energy and Industrial Strategy

Ten local policies were identified for appraisal, published between March 2011 and June 2017. The policies were authored by three different organisations (Additional material Appendix 2). The Southampton City Council led or was involved in the development of all local policies. Southampton City Clinical Commissioning Group co-authored three policies (30%) and Southampton Safe City Partnership authored one (10%).

4.2 Policy appraisal findings - national

Figure 2 shows the frequency of the priority areas and policy options across the 57 national policies. The priority areas that support public health processes were frequently included. The evidence-based practice and strengthening the evidence base through research and evaluation policy options featured in 79% (n=45) and 61% (n=35) of the national policies respectively. Community participation and accountability frameworks both featured in 56% (n=32) of the national policies. However, managing conflicts of interest was the second least frequent policy option (n=7, 12%). Multi-stakeholder collaboration was mentioned in more than half (n=33, 58%) of the national policies but multisectoral policy development was less common (n=11, 19%).

The action-orientated priority areas were less frequently included than those supporting good public health processes, with child health having the lowest prevalence (Figure 2). Fewer than half (n=27, 47%) of the policies included one of the four infants/children policy options. Complementary feeding initiatives was the least common policy option, appearing in only 9% (n=5) of national policies, while children's food marketing restrictions featured in 16%

For peer review only

(n=9). More than three quarters (n=46, 81%) of the policies contained one of the seven maternal policy options. Maternal nutrition related policy options (n=11, 19% to n=26, 46%) were better represented than those relating to maternal physical activity (n=15, 26% to n=16, 28%). One third (n=20, 35%) of policies contained strategies to strengthen health systems. The need for workforce development in behaviour change strategies was cited in 32% (n=18) of policies but workforce development in empowerment approaches was rarely mentioned (n=8, 14%).

4.2.1 Policy content according to policy type

The frequencies of each policy option for the three national policy types are presented in Figure 3. Among the eight **acts and codes**, nutrition labelling was the most common policy option appearing in 50% (n=4). Restrictions on marketing foods and beverages to children and evidence-based practice featured in 38% (n=3), while product reformulation, managing conflicts of interest and multisectoral collaboration were mentioned in 25% (n=2). Six policy options did not feature in any of the acts or codes. The vast majority of the 22 **white papers** included evidence-based practice and community participation (both n=18, 82%) and 73% (n=16) featured accountability frameworks and multisectoral collaboration. While nutrition in public institutions was the most common action-orientated policy option (n=11, 50%), developing workforce empowerment skills was not cited in any white papers and complementary feeding guidance was mentioned in only one (5%). Most of the 27 **evidence-based guidance** documents included evidence-based practices (n=24, 89%) and strengthening the evidence base through research (n=20, 74%). Nutrition guidelines featured in almost two thirds (n=17, 63%) but managing conflicts of interest was mentioned in only one (4%).

4.2.2 Policy content according to ecological model of health

For peer review only - http://bmjopen.bmj.com/site/about/guidelines.xhtml

Policy options to improve the macro environment were featured in half of the national policies (n=33, 58%). Policy options to create supportive social and physical environments in organisations featured more frequently (n=37, 65%), while those targeting individual level behaviour change were most common (n=41, 72%). Cochran's Q test revealed that there were no statistically significant differences (p=0.1) in representation of the ecological levels across the national policies.

4.3 Policy appraisal findings - local

Figure 4 shows the frequency of each policy option (including the three additional urban planning policy options) across the 10 local policies. Most of the local policies cited evidence-based practice (n=8, 80%), multisectoral collaboration (n=7, 70%) and multisectoral policy development (n=5, 50%). Maternal physical activity related policy options had a strong representation with physical activity guidance, urban planning, improving green spaces and physical activity facilities, and improving active transport infrastructure all appearing in 50% (n=5) of the local policies. Nutrition-focused policy options featured less frequently with five maternal nutrition options featuring in 10% (n=1) or less of the local policies. The two workforce development policy options also featured in only 10% (n=1) of local policies. Analysis against the ecological model of health revealed that macro environmental policy options featured in 90% (n=9) of the local policies, those targeting individual level behaviour change appeared in 50% (n=5), and those supporting healthy organisational settings in 30% (n=3). Cochran Q test revealed that there was a statistically significant difference in representation of these ecological levels across the local policies (p=0.02).

5. Discussion

For peer review only

5.1 Summary of Results

Among the 57 national and 10 local policies identified in this study, policy options supporting public health processes were adopted more frequently than the action-orientated options targeting maternal and child dietary and physical activity behaviours. This finding was consistent across the three types of national policies and the local policies, although some differences were apparent. Multisectoral policy development was more common among the local than the national policies, while using targets to monitor policy implementation was more frequently included in the national policies. Managing conflicts of interest was rarely considered in the white papers or evidence-based guidelines but was addressed more often in the acts and codes, and local policies.

For the action-orientated policy options, those with a nutrition focus were more frequent than the physical activity ones among the national policies. Acts and codes in particular did not contain any physical activity policy options. The opposite was observed for local policies where there was a heavy focus on urban planning strategies to promote physical activity. Complementary feeding guidance was virtually missing from all types of national policies, while nutrition labelling did not appear in any local policies. The need for workforce development in behaviour change skills was recognised across the white papers, evidence-based guideline and local policies, but workforce training in empowerment or self-care approaches was only cited in evidence-based documents and one local policy.

5.2 Comparison with Literature

This policy review is unique, compared with previous assessments of government action on obesity and NCDs in its focus on policy options specific to maternal, infant and child health. Existing policy frameworks consistent with the WHO Action Plan consider the entire population and measure progress for a specific behaviour or health outcomes. For example,

For peer review only - http://bmjopen.bmj.com/site/about/guidelines.xhtml

the World Cancer Research Fund International NOURISHING framework and the International Network for Food and Obesity Research, Monitoring and Action Support (INFORMAS) Healthy Food Environment Policy Index (Food-Epi) considered policy activities for healthy eating and food environments,[23] while the WHO Global Monitoring Framework focuses on the prevalence of NCD risk factors.[17] Policy options that support the adoption of healthy nutrition and physical activity behaviours prior to conception (including adolescence) and during pregnancy, infancy and childhood have been recognised by the UK Chief Medical Officer as critical to prevent NCDs in future generations and help curb healthcare costs.[19]

The results of this study identified that nationally, policy options related to maternal health were more frequently adopted than those related to child and infant health. General nutrition guidance and nutrition labelling were among the most frequent action-oriented policy options across the three types of national policies. Research from Thailand and England that used the Food-Epi framework similarly found good implementation of dietary guidelines and nutrition labelling when compared with international examples of good practice.[24 25] However, our study additionally assessed the inclusion of complementary feeding guidelines which we found to be the least well adopted policy option. Complementary feeding is important for child development, growth and health, and this period of life presents a window of opportunity to prevent malnutrition in all forms.[26] Over the past few decades progress on the inclusion of policy options to promote breastfeeding has been made but this has not been matched by efforts to support complementary feeding, as shown by our study's results. Other countries have also shown limited success in meeting WHO complementary feeding recommendations[27 28] indicating that government action on this issue is needed.

A systematic review of the determinants of poor nutrition in children aged below 3 years identified that effective intervention strategies include improving maternal self-efficacy,

For peer review only - http://bmjopen.bmj.com/site/about/guidelines.xhtml

being of sufficient intensity and reaching mothers during pregnancy.[29] Building the capacity of health professionals through workforce development is thus essential to engagement and supporting behaviour change.[30] A review of policy options to address obesity in Spain identified workforce development in obesity prevention as one of the most popular approaches for governments to adopt.[31] The results of our policy review showed that workforce development in behaviour change skills was included in national English policies. However, recognition of the need to up-skill healthcare professionals in empowerment approaches that improve self-efficacy was not included in any white papers, acts or codes. There is growing evidence that information is not enough to prompt behaviour change, particularly among disadvantaged groups, and that media campaigns increase inequalities in health behaviours.[32-34] While a number of the evidence-based documents reviewed in this study did include workforce empowerment approaches, the lack of coverage in white papers indicates that evidence is not adequate for policy option adoption. Policy decision-making results from a combination of factors including the framing of an issue, political ideology, evidence of effectiveness and cost-effectiveness, timing and 'groundswell' or societal attitudes.[9 13]

Recent reviews of obesity-related policy analyses identified an overwhelming focus on national or state/provincial governments, and a lack of consideration for how policy option adoption varies between national and local governments.[13 18] While limited to only one local authority, our study identified a clear difference in the focus of national and local policies. National documents contained a greater number of nutrition-related policy options, while local documents covered more policy options for physical activity, particularly macro-environmental options. The transition of public health into local authorities in the UK has provided increased opportunities for health considerations in planning and environment policies.[35] The dominance of physical activity over dietary policy options in Southampton

For peer review only - http://bmjopen.bmj.com/site/about/guidelines.xhtml

mirrors attitudes across other local councils in England. A consultation with staff from 14 councils identified low awareness of policy options for creating healthy food environments, but good knowledge of planning activities to improve cycling and walking, and access to open spaces.[36] These findings suggest that the conditions for policy action, including public and political value, resources and operational capabilities [37] are each being met for physical activity strategies but not yet for population level nutrition strategies.

There is a call for governments to enact regulation to create healthy food and physical activity environments as these policy options are consistently effective at improving lifestyle choices and do not widen inequalities in the way that information strategies can.[16] Our study showed that policy options to improve the macro environment were less well adopted by the English government than those targeting organisational settings or individual level behaviour change, although this difference was not statistically significant. Reviews that assessed international actions to address obesity across low, middle and high income countries similarly identified limited appetite from national governments for regulatory action and a preference for educational strategies.[38 39] The introduction of a UK sugar-sweetened beverage levy in 2018 indicates a growing global trend, led by action from governments in Mexico, France and Denmark, for stronger government actions to address obesity. However, concerns about adequate enforcement and the effectiveness of self-regulatory and voluntary codes, such as those used to govern restrictions for online advertising of unhealthy food to children or the reformulation of sugar and salt in foods, indicate that tougher regulatory action in these areas is still needed.[38]

Similar to reviews of the implementation of national policies related to food environments in New Zealand, Thailand and England,[24 25 40] we found that policy options supporting sound public health processes were frequently included across policy types. However, this study additionally identified that only a small proportion of English policies contained details about

For peer review only

managing conflicts of interest. This issue is important for governments to consider during policy development and adoption because strategies to improve maternal and child dietary and physical activity behaviours could impact upon a number of profit-driven commercial industries. In particular, commercial interests from infant formula manufacturing companies directly compete with breastfeeding.[41] Strategies to manage conflicts of interest were not included in a number of policies where potential conflicts are likely including The Processed Cereal–based Foods and Baby Foods for Infants and Young Children (England) Regulations. Limiting commercial influence and developing clear processes for accountability of both the public and the private sector are necessary.[23]

5.3 Implications for policy and research

The UK Government's current childhood obesity strategy outlined few measures for early childhood despite the WHO's Commission on Ending Childhood Obesity recommending member states develop clear complementary feeding guidance, including portion sizes and recommendations to avoid sugar sweetened beverages and energy-dense nutrient-poor foods. The much-awaited review by the Scientific Advisory Committee on Nutrition on complementary feeding will create an opportunity for the publication of clear guidelines in national white papers and their adoption by local and national agencies.

Health and social care services that support behaviour change by empowering individuals to generate their own solutions have shown beneficial effects on the self-efficacy of women from disadvantaged populations.[42] Practitioners from a range of backgrounds can be successfully trained in empowerment skills and these offer great potential to be used in routine health and social care to support behaviour change among harder to reach women and families.[43] The NHS 'making every contact count' movement recognises these opportunities for contact but further leadership is required from national government to include this policy

For peer review only

option in white papers and systematically build workforce capacity in empowerment skills across England.

Leadership and funding from state and national governments has been shown to affect policy priorities at the local level. Local governments in the State of Victoria in Australia have higher adoption of policy initiatives to support physical activity and healthy eating than other states. This increased activity has been directly attributed to the mandatory requirement under the Victorian Health Act 1958 and the Planning and Environment Act 1987 for local governments to prepare Municipal Public Health Plans, in addition to the offer of financial incentives.[9 44] In the UK, The National Planning Policy Framework requires planning departments to promote healthy communities, taking into account and supporting local Joint Health and Wellbeing Strategies. This national leadership has shown to be beneficial for physical activity related policy options but local public health teams now need to work strategically with planning and environment colleagues to support more healthy eating opportunities including zoning restrictions of takeaways or licensing requirements for the sale of healthy foods.[36] Collaborations between local universities and public health teams could provide necessary evidence for local government action to support development of healthy food environments, as local evidence provides impetus for local government action [9 45]. Similarly, supranational unions, such as the European Union (EU), have provided leadership or hindrance for member states directing initiatives related to the analysis conducted in this study. While it was beyond the scope of this study to assess EU governance over English policies, Brexit provides opportunities for brave policy change that are supportive of healthy dietary and physical activity behaviours.

Recent evidence from a leaked email shows that Coca Cola placed indirect pressure on governments through an industry-funded scientific institute to influence policy to benefit commercial companies rather than the public.[46] Public confidence can be damaged if

For peer review only - http://bmjopen.bmj.com/site/about/guidelines.xhtml

government's management of conflicts of interest is not sufficiently documented.[47] By using the international best practice standards for managing conflicts of interest, governments in England could appropriately manage this issue going forward.[48]

5.4 Strengths and Limitations

This study is one of few policy reviews to focus on maternal, infant and child health for NCD prevention and to consider government activity at both the national and local levels. The study involved a comprehensive desktop search for three types of policies that was aided by senior health officials in local and national government to enhance completeness of the search strategy. Strengths of the appraisal framework include that it mirrored current international best practice standards, was developed and tested by three researchers independently. For quality assurance, one author completed the data extraction for all identified policies and half were also coded by a second researcher.

This study also had a number of limitations. Some policies may have been missed because they were not available electronically or were not identified by our search. The local policy results for Southampton may not be representative of action in other local authorities. In addition, the appraisal framework and data extraction results were based on secondary data analysis only. They were not validated through extensive consultation with policy makers and other expert stakeholders due to resource limitations. However, key search terms and inclusion and exclusion criteria for each policy option were used to increase accuracy of the document assessment. Furthermore, the use of a dichotomous measure does not provide comprehensive details about the scale of policy option implementation and not all policy options would be expected to appear in every policy. However, adopting a quantitative approach enabled assessment of overall adherence to WHO international best practice and compared the level of activity between priority areas allowing easy identification of areas for

For peer review only

improvement. This approach could be used by governments to monitor progress on NCD prevention targeting women and children.

6. Conclusion

This study assessed policies relevant to improving maternal and child dietary and physical activity behaviours against the WHO international standard. It aids understanding of the national and local progress in England of adopting policy options to prevent NCDs. Analyses of 57 national policies highlighted greater inclusion of policy options related to maternal than child health (particularly complementary feeding initiatives) and individually focused policy options (such as nutrition labelling) than environmental strategies. Action at the local level showed a stronger focus on policy options for physical activity than those for healthy eating, and for child health than for maternal health. Policy options that underpin good public health processes such as evidence-based strategies and multisectoral collaboration were widely adopted by both national and local governments, but further action is needed by policy makers to manage conflicts of interest and curb industry lobbying during policy development. Opportunities exist for the research community and health professionals to influence policy in a meaningful way. Such opportunities include forming coalitions to mobilise societal support and framing issues as solutions in a way that is palatable to policy makers. The timing of external events can positively influence policy decision making. The 2012 Olympics provided impetus for policy action supportive of physical activity.[49] Challenging as the management of Brexit is, it provides a unique opportunity for policy change that holds great potential to reform food and transport systems to be supportive of healthy dietary and physical activity behaviours.

For peer review only

For peer review only - http://bmjopen.bmj.com/site/about/guidelines.xhtml

Acknowledgements

We are grateful to Patsy Coakley for computing support and to the programme manager at Public Health England for their support with policy identification. We thank our funders for their support and would like to acknowledge that the views expressed in this publication are those of the authors and not necessarily those of the funding organisations.

Source of funding

This work was supported by funding from:

- NIHR Southampton Biomedical Research Centre, University of Southampton University Hospital Southampton NHS Foundation Trust
- European Union's Seventh Framework Programme (FP7/2007-2013), project EarlyNutrition (289346)
- CV was supported by a fellowship from the University of Southampton (PCTA36/2015)

Conflicts of interest statement

DPN, SShaw, DCongalton, DChase, HMI and CV have no conflicts of interest to declare. SStrommer, TR, WL, MB and JB have received grant research support from Danone Nutricia Early Life Nutrition. CC has has received consultancy, lecture fees and honoraria from AMGEN, GKS, Alliance for Better Bone Health, MSD, Eli Lilly, Pfizer, Novartis, Servier, Medtronic and Roche. The study described in this manuscript is not related to these conflicted relationships.

Contributor statement

DPN and CV conceived the study and wrote the first draft of the manuscript. HMI, JB, MB, DChase, CC and WTL contributed to the study design and helped draft the manuscript. DPN, TR, SStrommer and DChase identified the relevant policies. DPN, DCongalton and CV designed the policy audit tool. DPN, SShaw, DCongalton and CV conducted the policy audit. All authors read and approved of the manuscript.

Data sharing statement

The data are available upon request to the corresponding author.

For peer review only - http://bmjopen.bmj.com/site/about/guidelines.xhtml

References

1. Abajobir AA, Abate KH, Abbafati C, et al. Global, regional, and national comparative risk assessment of 84 behavioural, environmental and occupational, and metabolic risks or clusters of risks, 1990-2016: a systematic analysis for the Global Burden of Disease Study 2016. *The Lancet*;390(10100):1345-422.
2. World Health Organisation. *Noncommunicable Diseases (NCD) Country Profiles - United Kingdom*. Secondary Noncommunicable Diseases (NCD) Country Profiles - United Kingdom 2014. http://www.who.int/nmh/countries/gbr_en.pdf.
3. Barker DJ, Thornburg KL. Placental programming of chronic diseases, cancer and lifespan: a review. *Placenta* 2013;34(10):841-5.
4. United Nations Secretary-General. Global Strategy for Women's, Children's and Adolescent's Health (2016-2030). New York, 2015.
5. World Health Organisation. Comprehensive Implementation Plan on Maternal, Infant and Young Child Nutrition. Geneva, Switzerland, 2014.
6. World Health Organisation. Report of the Commission on Ending Childhood Obesity. Geneva, Swtizerland, 2016.
7. Swinburn BA, Sacks G, Hall KD, et al. The global obesity pandemic: shaped by global drivers and local environments. *Lancet* 2011;378(9793):804-14.
8. Department of Health UK. Healthy Lives, Healthy People: Our strategy for public health in England. London, 2010.
9. Allender S, Gleeson E, Crammond B, et al. Moving beyond 'rates, roads and rubbish': How do local governments make choices about healthy public policy to prevent obesity? *Australia and New Zealand Health Policy* 2009;6:20.
10. UK Parliament. *Acts*. Secondary Acts 2017. http://www.parliament.uk/about/how/laws/acts/.
11. UK Parliament. *White Papers*. Secondary White Papers 2017. http://www.parliament.uk/site-information/glossary/white-paper/.
12. National Institute for Health and Care Excellence (NICE). *What we do*. Secondary What we do 2017. https://www.nice.org.uk/about/what-we-do.
13. Clarke B, Swinburn B, Sacks G. The application of theories of the policy process to obesity prevention: a systematic review and meta-synthesis. *BMC Public Health* 2016;16(1):1084.
14. World Health Organisation. Global Action Plan for the Prevention and Control of Noncommunicable diseases 2013-2020,. Geneva: World Helth Organisation, , 2013.
15. Davies M, Macdowall W. *Health promotion theory*. Berkshire, UK,: Open University Press,, 2006.
16. Hyseni L, Atkinson M, Bromley H, et al. The effects of policy actions to improve population dietary patterns and prevent diet-related non-communicable diseases: scoping review. *Eur J Clin Nutr* 2017;71(6):694-711.
17. World Health Organisation. Global Status Report on Noncommunicable Diseases 2014. Genva, 2014.
18. Phulkerd S, Lawrence M, Vandevijvere S, et al. A review of methods and tools to assess the implementation of government policies to create healthy food environments for preventing obesity and diet-related non-communicable diseases. *Implementation science : IS* 2016;11:15.
19. Davies S. Annual Report of the Chief Medical Officer, 2014, The Health of the 51%: Women. London, UK, 2015.
20. Southampton City Council. Index of Multiple Deprivation 2015: An analysis of changes since 2010. Southampton 2016.
21. Mortimore A. Public Health Annual Report 2015. In: Coates B, King D, eds. Southampton, 2016.
22. Stata Statistical Software: Release 13 [program]. 11 version. Texas: College Station, 2013.
23. Vandevijvere S, Swinburn B, International Network for F, et al. Pilot test of the Healthy Food Environment Policy Index (Food-EPI) to increase government actions for creating healthy food environments. *BMJ Open* 2015;5(1):e006194.
24. Phulkerd S, Vandevijvere S, Lawrence M, et al. Level of implementation of best practice policies for creating healthy food environments: assessment by state and non-state actors in Thailand. *Public Health Nutr* 2017;20(3):381-90.
25. The Food Foundation. Food Environment Policy Index (Food-Epi) for England. London, 2016.
26. Reynolds CM, Gray C, Li M, et al. Early Life Nutrition and Energy Balance Disorders in Offspring in Later Life. *Nutrients* 2015;7(9):8090-111.
27. Carletti C, Pani P, Monasta L, et al. Introduction of Complementary Foods in a Cohort of Infants in Northeast Italy: Do Parents Comply with WHO Recommendations? *Nutrients* 2017;9(1).
28. White JM, Begin F, Kumapley R, et al. Complementary feeding practices: Current global and regional estimates. *Matern Child Nutr* 2017;13 Suppl 2.

For peer review only

29. Summerbell C, Moore H, O'Malley C. Consequences and determinants of poor nutrition in children aged 0-3 years, and public health interventions that may improve dietary intake: a general review. *Journal of Children's Services* 2014;9(2):128-42.
30. Baillie E, Bjarnholt C, Gruber M, et al. A capacity-building conceptual framework for public health nutrition practice. *Public Health Nutrition* 2009;12(8):1031-38.
31. Gonzalez-Zapata LI, Ortiz-Moncada R, Alvarez-Dardet C. Mapping public policy options responding to obesity: the case of Spain. *Obes Rev* 2007;8 Suppl 2:99-108.
32. Kelly MP, Barker M. Why is changing health-related behaviour so difficult? *Public Health* 2016;136:109-16.
33. Beauchamp A, Backholer K, Magliano D, et al. The effect of obesity prevention interventions according to socioeconomic position: a systematic review. *Obes Rev* 2014;15(7):541-54.
34. Lorenc T, Petticrew M, Welch V, et al. What types of interventions generate inequalities? Evidence from systematic reviews. *Journal of Epidemiology & Community Health* 2013;67(2):190-3.
35. Middleton J. Time to put health at the heart of all policy making. *BMJ* 2017;357:j2676.
36. Town and Country Planning Association. Building the foundations: Tackling obesity through planning and development. London, 2016.
37. Moore MH. *Creating public value : strategic management in government*. Cambridge, Mass.: Harvard University Press, 1995.
38. Roberto CA, Swinburn B, Hawkes C, et al. Patchy progress on obesity prevention: emerging examples, entrenched barriers, and new thinking. *Lancet* 2015;385(9985):2400-9.
39. Lachat C, Otchere S, Roberfroid D, et al. Diet and physical activity for the prevention of noncommunicable diseases in low- and middle-income countries: a systematic policy review. *PLoS Med* 2013;10(6):e1001465.
40. Vandevijvere S, Mackay S, Swinburn B. Benchmarking Food Environments: progress by the New Zealand government on implementing recommended food environment policies and prioritised recommendations. Auckland 2017.
41. Wright CM, Waterston AJR. Relationships between paediatricians and infant formula milk companies. *Archives of Disease in Childhood* 2006;91(5):383-85.
42. Baird J, Jarman M, Lawrence W, et al. The effect of a behaviour change intervention on the diets and physical activity levels of women attending Sure Start Children's Centres: results from a complex public health intervention. *BMJ Open* 2014;4(7):e005290.
43. Lawrence W, Black C, Tinati T, et al. 'Making every contact count': Evaluation of the impact of an intervention to train health and social care practitioners in skills to support health behaviour change. *J Health Psychol* 2016;21(2):138-51.
44. Yeatman H. Action or inaction? Food and nutrition in Australian local governments. *Public Health Nutr* 2009;12(9):1399-407.
45. Middleton G, Henderson H, Evans D. Implementing a community-based obesity prevention programme: experiences of stakeholders in the north east of England. *Health Promot Int* 2014;29(2):201-11.
46. Sacks G, Swinburn BA, Cameron AJ, et al. How food companies influence evidence and opinion – straight from the horse's mouth. *Critical Public Health* 2017:1-4.
47. Newton A, Lloyd-Williams F, Bromley H, et al. Food for thought? Potential conflicts of interest in academic experts advising government and charities on dietary policies. *BMC Public Health* 2016;16(1):735.
48. World Health Organisation. Addressing and managing conflicts of interest in the planning and delivery of nutrition programmes at country level. Geneva, Switzerland, 2016.
49. Piggin J, Hart L. Physical activity advocacy in the UK: a multiple streams analysis of a hybrid policy issue. *Leisure Stud* 2017;36(5):708-20.

For peer review only

List of figure legends

Figure 1: The schematic diagram of the policy identification process

Figure 2: Percentage of all 57 national policy documents for which each policy option in the policy appraisal framework was present

Figure 3: Percentage of the 8 acts and codes, 22 white papers and 27 evidence-based guidance documents for which each policy option in the policy appraisal framework was present

Figure 4: Percentage of the 10 local policy documents for which each policy option in the policy appraisal framework was present

For peer review only

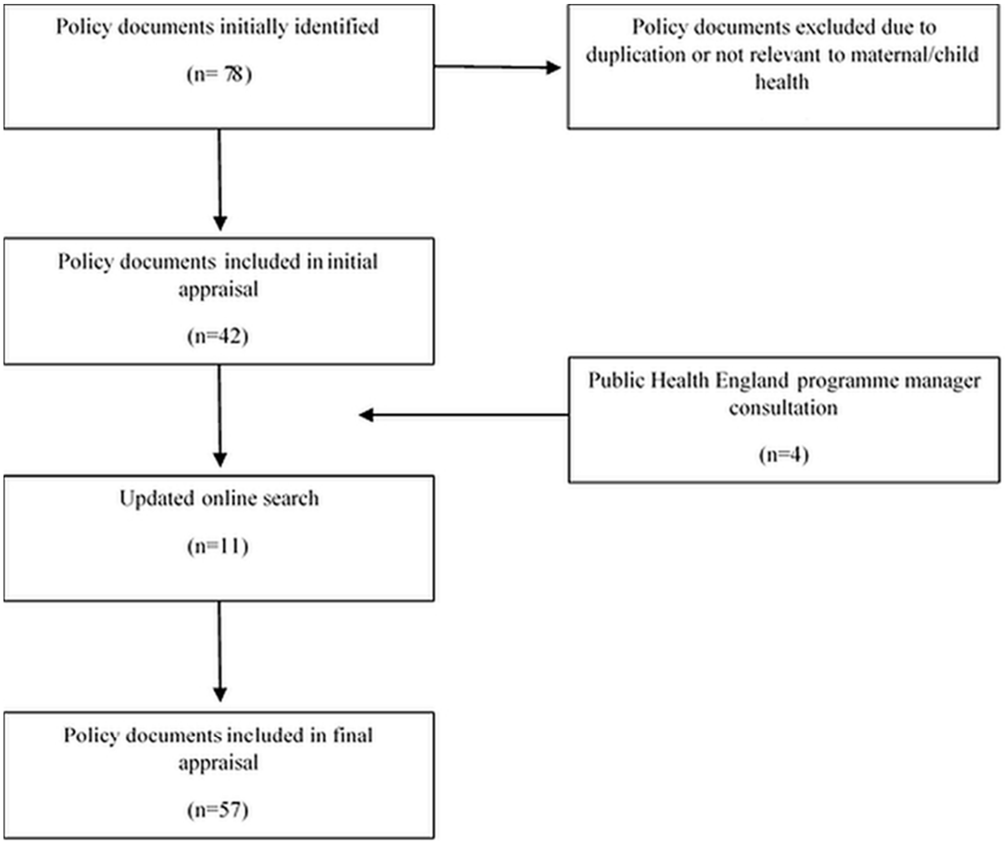

The schematic diagram of the policy identification process

32x27mm (600 x 600 DPI)

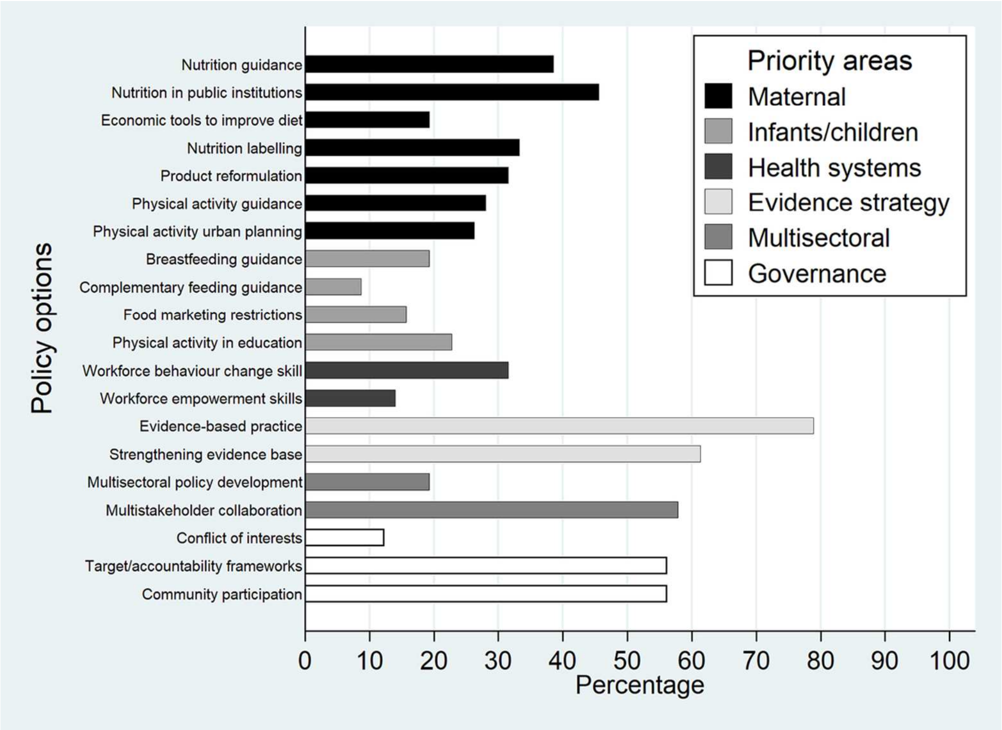

Percentage of all 57 national policy documents for which each policy option in the policy appraisal framework was present

105x76mm (300 x 300 DPI)

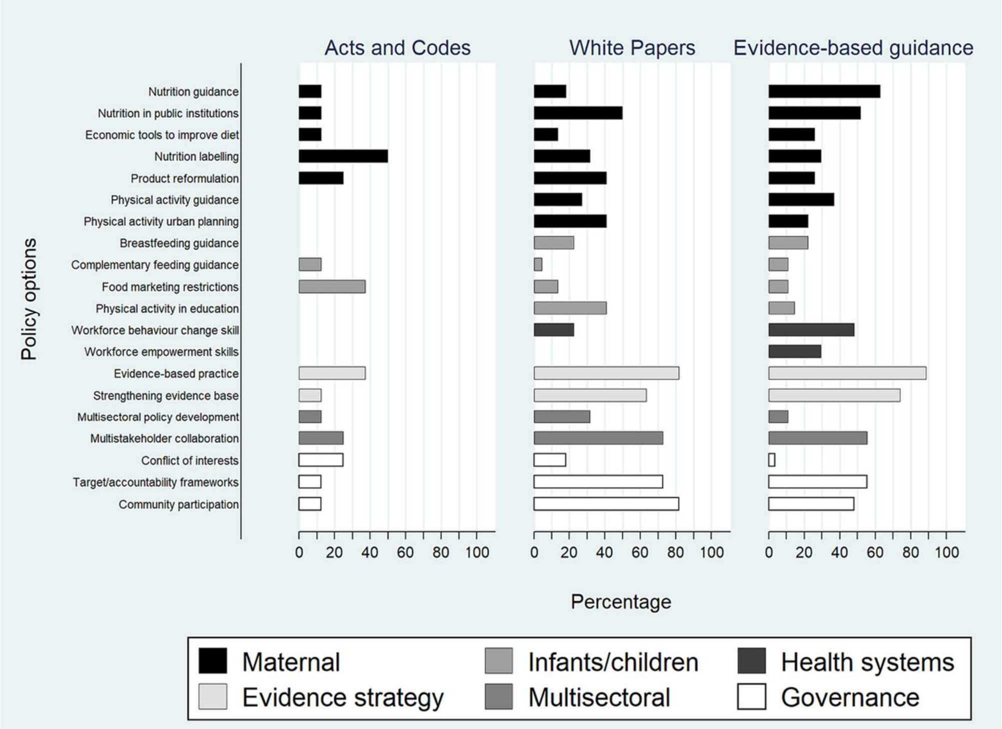

Percentage of the 8 acts and codes, 22 white papers and 27 evidence-based guidance documents for which each policy option in the policy appraisal framework was present

105x76mm (300 x 300 DPI)

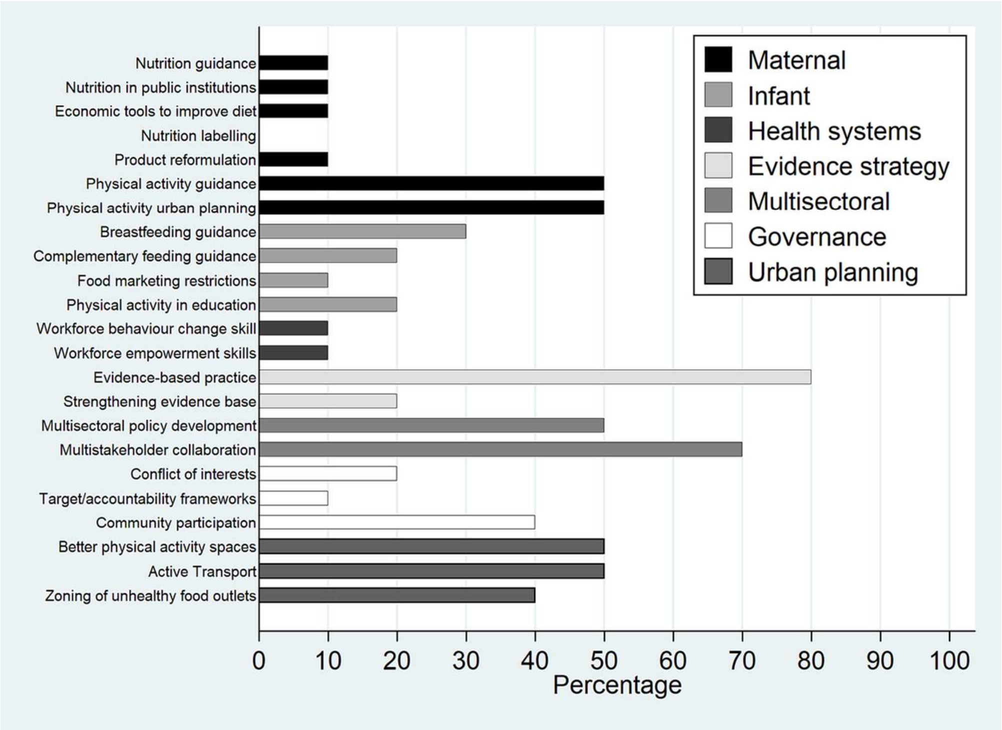

Percentage of the 10 local policy documents for which each policy option in the policy appraisal framework was present

105x76mm (300 x 300 DPI)

Additional material

Appendix 1: The Policy Appraisal Framework used to analyse the policy documents identified

Priority Areas	Policy options	Detail	Search terms for data extraction	Inclusion and Exclusion criteria[a,b]
**Maternal health** *Objective 3: Reducing modifiable risk factors Overarching principle : life-course approach*	1. Social media campaigns and/or dietary guidance	39.8	social media, nutrition, education, healthy eating, health promotion, social marketing, diet, food, guidelines, guidance, healthy lifestyle, mass media, campaigns, health promotion, eat well plate, 5 a day.	**Inclusion:** Makes reference to dietary education and/or materials including dietary guidelines or intent to develop dietary guidelines. Includes material specifically for adolescent females (preconception health). Includes reference to external guidance linked in the policy document that meet the criteria, as long as the external guidance is accurately referenced in the correct place in the policy document. **Exclusion**: Education initiative solely focusing on children's diets, breastfeeding (except diets for breastfeeding women), alcohol consumption and smoking.
	2. Healthy food and health promotion in public institutions	39.5, 39.9, 41.4 (iv)	public institute, standard, guideline, healthy environment, active transport, government agencies, food, nutrition, physical activity exercise, movement, school, hospital, meals.	**Inclusion:** Reference to guidelines/health promotion of food, diet and physical activity health promoting environments within public institution. Includes reference to schools, hospitals and any government agencies. **Exclusion:** Smoke-free, alcohol and breastfeeding environments.
	3. Economic tools to increase healthy and reduce less healthy food consumption	39.6 30.2	taxes, subsidies, sugar sweetened beverages, fiscal strategies, fruit and vegetable, finance, vouchers.	**Inclusion:** Must make reference to intent to use fiscal measures to increase consumption of healthy foods and reducing consumption of less healthy foods. Includes pricing initiatives in schools, hospitals, leisure centres. **Exclusion:** Physical activity or public transport subsidies.

For peer review only - http://bmjopen.bmj.com/site/about/guidelines.xhtml

	4. Nutrition labelling	39.10	food labelling, nutrition labelling, packaged food, front of pack, back of pack, traffic lights, health claims, nutrition information panel	**Inclusion:** Must make reference to nutrition information or improving health. Labelling on pre-packaged foods or nutrition facts. Includes nutrition prompts about foods provided by an establishment, e.g catering in schools, hospitals, leisure centres. Nutrition labelling for infants and babies including baby formula is included. **Exclusion:** Reference to food safety.
	5. Guidelines to reduce sodium, saturated fat, free sugar and increase fruit and vegetables for manufacturers to create healthy agriculture, retail and catering sectors	39.3, 39.4, 39.7	sodium, salt, trans (fat) saturated fat, (free) sugars, portion/serve size, fruit and vegetables, reformulation. Agriculture, farming, food retailers, caterers, subsidies, land availability, energy density.	**Inclusion:** Product reformulation and the improvement of the nutritional quality of the food – specifically saturated/trans fat, sugars, salt, fruit and vegetables, energy density or portion sizes. Not required to directly refer to maternal health. Includes agricultural, manufacturing, retail and catering sectors. Includes reference to changing purchasing of a product with less salt, sodium, sugar, or fat of catering in schools, hospitals, leisure centres etc.
	6. Physical activity guidelines and/or public campaigns	41.4 (iii), 41.4 (v), 41.5, 41.1	mass media, physical activity, inactivity, sedentary, active transport, exercise, healthy lifestyle, mass media, social media, leaflet, booklets,	**Inclusion:** Reference to guidelines/recommendations for physical activity, exercise and active transport or ways to reduce inactivity. Includes intent to develop physical activity guidelines. Includes material regarding adolescent females (preconception health). **Exclusion:** Education initiatives solely focusing on children.
	7. Urban, transport and recreation planning to increase daily activity	41.4 (i), 41.4 (iv)	urban planning, urban transport, recreation, infrastructure walking, cycling, safety, active transport, green space, natural environments, lockers, facilities, shower.	**Inclusion:** Improving physical activity and active transport facilities including accessibility, availability, affordability and safety. Includes community playgrounds and outdoor spaces. Includes initiatives to encourage active transport leading to improved physical activity. **Exclusion:** Initiatives to enhance social connectivity.

For peer review only - http://bmjopen.bmj.com/site/about/guidelines.xhtml

Urban Planning [c]	8. Green Spaces and physical activity facilities	41.4 (iv)	green Spaces, playgrounds, community gyms, physical activity, sports, parks	**Inclusion:** Increasing numbers or improving community green spaces and the availability of council-led physical activity facilities that enable people to be physically active.
	9. Active Transport	41.4<br>41.4 (i)	traffic safety, walking cycling, footpath, pathway, active transport	**Inclusion:** Safety in terms of traffic lights and street lights and safety to enable active transport including footpaths and any sort of pathway and availability of community bikes. Explicit reference to changes at a local policy level that improve the opportunity for active transport, e.g. changes to infrastructure and physical environmental improvements.
	10. Licensing or Zoning of less healthy food outlets	39.7<br>39.4	zoning, takeaway outlets, diary, corner store	**Inclusion:** Licensing and zoning to regulate the density of less healthy food outlets.
**Infant health**<br><br>*Objective 3: Reducing modifiable risk factors Overarching principle : life-course approach*	1. Breastfeeding guidelines/ initiatives	39.1, 18.5, Annex 43(i)	breastfeed, nursing	**Inclusion:** Recommendations for breastfeeding and reference made to breastfeeding recommendations or action plans. Includes promotion of breastfeeding friendly environments and encouragement or promotion of breastfeeding. Includes cross-reference to breastfeeding guidelines and links to reputable sources with explicit mention of the criteria.<br>**Exclusion:** Food and dietary guidelines for breastfeeding women.
	2. Complementary feeding guidelines/ initiatives	39.1	complementary feeding, introducing solids, weaning	**Inclusion:** Reference to recommendations/ guidelines for complementary feeding and/or introducing solids, including infant and toddlers dietary guidelines.
	3. Guidelines/ restrictions for the marketing of food and beverages to children	33, 38, 39.2, 43.1	marketing, advertising, promotions, sponsorship	**Inclusion:** Details/restrictions of food marketing or advertising to children or reference made to the need for food marketing/advertising guidelines. Includes television, online, sponsorship of sports, school activities and other recreation activities. Also includes marketing not necessarily targeted at children but is intended for them, e.g. baby formula marketing.

For peer review only - http://bmjopen.bmj.com/site/about/guidelines.xhtml

	4. Physical activity opportunities in education institutions	41.4 (ii)	physical education, active movement, walking , cycling, physical activity, exercise, active transport, preschools, day care, Kindergartens, child care, outdoor play	**Inclusion:** Reference made to guidelines/ recommendations to physical activity options for education institutions. Includes requirements for making changes to environment to encourage physical activity. Includes initiatives external to school grounds but school-driven such as 'walking school bus'. Needs to refer to education institutions including pre-schools through to universities. **Exclusion:** Initiatives solely focusing on staff of these institutions.
**Strengthening health systems** *Objective 4 Overarching principle: Empowerment of people and communities*	1. Improving the knowledge, skills and motivation of the workforce (maternal and infant health professionals) through training in NCD and obesity prevention, specifically regarding behaviour change, diet and physical activity	48.4 (i, ii), 48.3(vii 30.8	skills, development, education, workforce develop, knowledge, training, behaviours skills	**Inclusion:** Regards training of health professionals inclusive of maternal and child health professionals to increase knowledge and skills in behaviour change, diet and/or physical activity requirements, inclusive of mothers (preconception, pregnancy and breastfeeding) and infants. Is about NCD prevention. **Exclusion:** Does not include smoking or alcohol consumption.
	2. Maternal and Infant health professionals empower people to manage their own condition and support family-centred/self-care, particularly among high-risk populations.	48.3 (vi),, 49.4 (iii)	empower, Patient/family/ community centre, self-care, cultural competent, cultural awareness, minority groups, low-income groups.	**Inclusion:** Reference to training of health professionals inclusive of maternal and child health professionals in skills that ensure the level of care is patient/family centred and/or empowering or a particular consideration for high risk populations. Not just about training but also competencies and training to adequate levels to provide empowerment. Includes empowerment of people who help target group, e.g. carers, parents. **Exclusion:** General person-centred approach statements.
**Evidenced based strategies** *Overarching principle*	1. Adoption of evidence based practice	18.7 32.1 39.8 41.5	best practice, evidence, strategies, evidence informed/based	**Inclusion:** Must refer to the use of evidence-base strategies (latest scientific evidence and/or best practice) to inform the development and implementation of policies/programmes /interventions relevant to diet and physical activity.

	2. Strengthen evidence base for effective dietary and physical activity interventions through research and evaluation	53.5<br>41.6	research, evaluation, scientific, knowledge base, best practice	**Inclusion:** Refers to research and evaluations of interventions/programmes to improve diet and/or physical activity. Includes the documents referring to the need for review or development guidance.
**Multisectoral action**<br>*Objective 2 Overarching principle*	1. Multisectoral development of the policy	18.4<br>27<br>29<br>30.5<br>30.6	*searched the policy authors and the introduction of the policy*.	**Inclusion:** Reference to the process of policy development. Must refer to more than one government department/government funded institution in policy development.
	2. Multistakeholder collaboration and cooperation	21.3<br><br>39.3<br>41.3	collaboration, co-operation, engagement, stakeholder, community, local government.	**Inclusion:** Development of collaborations /engagements with stakeholders for interventions/programme/policies. Includes stakeholders from multiple sectors, such as NGOs, industry, civil society groups, professional bodies, different disciplines, etc.<br>**Exclusion:** Not required to be directly related to diet/food and physical activity behaviour change. Documents cannot simply include multistakeholder action as a throwaway comment but must name examples.
**Governance and accountability**<br>*Objective 2 Overarching Principle: Management of real, perceived or potential conflicts of interest*	1. Management or acknowledgement of conflicts of interest	38<br><br>18.9	conflicts of interest, conflicting industries/ bodies, funding sources, guidelines	**Inclusion:** Reference to the explicit awareness of managing conflicts of interest when developing diet, physical activity or food/physical activity environment initiatives.<br>**Exclusion:** Initiatives related to smoking and alcohol consumption.
	2. Set targets/ establish accountability frameworks for NCDs, diet or physical activity behaviours	29<br>30.7<br><br>Pg. 5. Voluntary Global Targets	target, framework, accountability, plan, KPI's, indicators, outcomes, outputs, goal, objective, vision	**Inclusion:** Must refer to a framework or targets set by WHO or national/local governments that relate to NCDs, obesity, diet and physical activity. Includes intention to develop a framework or targets.<br>**Exclusion:** Accountability frameworks for policies that do not explicitly relate to NCD prevention i.e. professional code of conduct accountability frameworks.

For peer review only - http://bmjopen.bmj.com/site/about/guidelines.xhtml

	3. Implement participatory, community based approaches	30.10<br>48.1 (ii)	participation, community, empowerment, involvement, capacity building, consultation.	**Inclusion:** Reference to community members/ representative groups involvement in the development/ implementation of interventions/programmes and policy development. Must recognise that the community have a key role to play, not just about guidelines but how the local community are involved.

[a] It is acceptable for links to external documents to be included as a positive result for the different policy options. However the external documents must be linked to in the body of the text of the policy document being appraised and must the body of text must explicitly mention the policy option area being explored.

[b] Focusses on the physical components of health related to physical activity and diet, therefore doesn't include mental health in this instance.

[c] This section only applies to the Local policies from the case study.

[d] The colouring corresponds to the ecological model of health levels: light grey is individual behaviour change, grey is supportive social and physical environments in institutions, and dark grey is macro environmental policy.

For peer review only

Additional material

Appendix 2: List of all policy documents appraised arranged by policy type

**Policy Document Title**	**Date Published**	**Author**
Acts and codes		
The Processed Cereal–Based Foods and Baby Foods for Infants and Young Children (England) Regulations	December 2003	Department of Health
The Childcare Act 2006	July 2006	Ministry of Justice
The Infant Formula and Follow-on Formula (England) Regulations	December 2007	Department of Health
The UK Code of Non-Broadcast Advertising, Sales Promotion and Direct Marketing	August 2010	Advertising Standards Authority Ofcom
The UK Code of Broadcast Advertising (BCAP code)	August 2010	Advertising Standards Authority Ofcom
Health and Social Care Act 2012	June 2012	Department of Health
Children and Families Act 2014	March 2014	Department for Education<br>Ministry of Justice<br>Department for Business, Innovation & Skills[a]<br>Department for Work and Pensions<br>Department of Health
The Food Information Regulations 2014	July 2014	Department for Environment, Food and Rural Affairs
White papers		
Healthy Weight Healthy Lives	January 2008	Department of Health<br>Department for Children, Schools and Families[b]
Healthy Lives, Brighter Futures: the strategy for children and young people's health	February 2009	Department of Health<br>Department for Children, Schools and Families[b]
Healthy Lives Healthy People: our strategy for public health in England	November 2010	Department of Health
Health Visitor Implementation Plan 2011-2015: a call to action	February 2011	Department of Health
Supporting Families in the Foundation Years	August 2011	Department for Education<br>Department of Health
Joint Strategic Needs Assessment and Joint Health and Wellbeing Strategies Explained	December 2011	Department of Health
National Planning Policy Framework	March 2012	Department for Communities and Local Government

For peer review only - http://bmjopen.bmj.com/site/about/guidelines.xhtml

An Update in the Government's Approach to Tackling Obesity	July 2012	National Audit Office
Improving Children And Young People's Health Outcomes: a system wide response	February 2013	Department of Health
Giving all Children a Healthy Start in life	March 2013	Department for Education<br>Department of Health
Developing a Specification for Lifestyle Weight Management Services	March 2013	Department of Health
National Health Visitor Plan: progress to date and implementation 2013 onwards	June 2013	Department of Health<br>NHS England<br>Public Health England<br>NHS Health Education England
Briefing on the Government's Ambition for Cycling	August 2013	Department for Transport
Moving More, Living More: Olympic and Paralympic Games legacy	February 2014	Cabinet Office<br>Department of Health<br>Department for Digital, Culture, Media and Sport,<br>Department for Education<br>Mayor of London
Maternity and Early Years: making an good start to family life	March 2015	Department of Health
The Governments Responsibility Deal	March 2015	Department of Health
Statutory Guidance on Joint Strategic Needs Assessments and Joint Health Wellbeing Strategies.	March 2015	Department of Health
A Plan for Public Procurement: food & catering - balanced scorecard for public food procurement	July 2015	Department for Environment, Food and Rural Affairs
Five Year Forward View	October 2015	NHS England
Public Health Outcomes Framework, 2013-2016 (1A and 1B)	November 2015	Department of Health
Childhood Obesity: a plan for action	August 2016	Department of Health
Soft Drinks Industry Levy	December 2016	HM Revenue and Customs<br>HM Treasury
Evidence-based guidance documents		
Postnatal Care up to 8 Weeks After Birth	July 2006	NICE
Obesity Prevention	December 2006	NICE
Food Standards Agency Nutrient and Food Based Guidelines for UK Institutions	October 2007	Food Standards Agency

For peer review only

Behaviour Change: general approaches	October 2007	NICE
Antenatal Care for Uncomplicated Pregnancies	March 2008	NICE
Everybody Active, Every Day: an evidence-based approach to physical activity	October 2014	Public Health England
The Link Between Pupil Health and Wellbeing and Attainment: a briefing for head teachers, governors and staff in education settings	November 2014	Public Health England
Behaviour Change: individual approaches	January 2015	NICE
Physical Activity: for NHS staff, patients, and carers.	March 2015	NICE
Weight Management Before, During and After Pregnancy	July 2015	NICE
Postnatal Care	July 2015	NICE
Maternal and Child Nutrition	July 2015	NICE
A Quick Guide to the Government's Healthy Eating Recommendations	August 2015	Public Health England
Obesity in Children and Young People: prevention and lifestyle weight management programmes	August 2015	NICE
Multiple Pregnancy: twin and triplet pregnancies	September 2015	NICE
Sugar Reduction: the evidence for action	October 2015	Public Health England
From Evidence into Action: opportunities to protect and improve the nation's health	October 2015	Public Health England
Obesity and the Environment: regulating the growth of fast food outlets.	November 2015	Public Health England<br>Local Government Association
Nutrition Support in Adults	November 2015	NICE
The Influence of Maternal, Foetal and Child Nutrition on the Development of Chronic Disease in Later Life	November 2015	Scientific Advisory Committee on Nutrition
Obesity in Adults: pre obesity in adults: prevention and lifestyle weight management lifestyle weight management programmes	January 2016	NICE
Building the Foundations: tackling obesity through planning and development	February 2016	Local Government Association<br>Town and Country Planning Association<br>Public Health England

For peer review only - http://bmjopen.bmj.com/site/about/guidelines.xhtml

Healthier Food Procurement	August 2016	Local Government Association
Antenatal Care	September 2016	NICE
Childhood Obesity: brave and bold action	November 2016	House of Commons Health Committee
Guide to Creating a Front of Pack (FoP) Nutrition Label for Pre-packed Products Sold Through Retail Outlets	November 2016	Department of Health<br>Food Standards Agency<br>Welsh Government<br>Northern Ireland Government<br>Food Standards Scotland<br>British Retail Consortium
Healthy Weight, Healthy Futures: local government action to tackle childhood obesity	February 2017	Local Government Association
Local Policies		
Local Transport Plan 3: strategy and implementation plan for Southampton.	March 2011	Southampton City Council
Supporting Carers in Southampton: a joint strategy for improvement 2011-2015	December 2012	Southampton City Council<br>Southampton City Clinical Commissioning Group
Southampton's Joint Health and Wellbeing Strategy: gaining healthier lives in a healthier city	January 2013	Southampton City Council<br>Southampton City Clinical Commissioning Group
Southampton Local Plan: compliance with national planning policy framework	June 2013	Southampton City Council
Southampton City Strategy 2014-2025	July 2014	Southampton City Council
Southampton Safe City Strategy 2014-17	July 2014	Safe City Partnership (Hampshire Constabulary, Southampton City Council and other representatives)
Our Vision for Future Southampton	June 2015	Southampton City Council
Health and Wellbeing Strategy 2017-2025	March 2017	Southampton City Council<br>Southampton City Clinical Commissioning Group
Cycling Southampton: a cycling strategy for our city 2017-2027	June 2017	Southampton City Council
Children and Young People's Healthy Weight Plan	June 2017	Southampton City Council

[a] superseded by Department for Business, Energy and Industrial Strategy, [b] superseded by Department of Education

For peer review only
